# Machine-Learning-Enabled Raman Spectroscopy Refines Indocyanine Green Fluorescence Boundaries for Precise Glioblastoma Margin Delineation

**DOI:** 10.34133/research.1216

**Published:** 2026-04-01

**Authors:** Dong Han, Kai Liu, Lijun Zhu, Yufei Miao, Jinglei Zhang, Ziyang Wang, Guangming Lu, Christopher J. Butch, Huiming Cai, Shuming Nie, Yiqing Wang

**Affiliations:** ^1^ Department of Biomedical Engineering, College of Engineering and Applied Sciences, State Key Laboratory of Analytical Chemistry for Life Science, Nanjing University, Nanjing, Jiangsu 210023, China.; ^2^ Department of Medical Imaging, Jinling Hospital, School of Medicine, Nanjing University, Nanjing, Jiangsu 210002, China.; ^3^ Nanjing Nuoyuan Medical Devices Co. Ltd, Nanjing, Jiangsu 211500, China.; ^4^ Department of Biomedical Engineering, University of Illinois at Urbana-Champaign, Urbana, IL 61801, USA.

## Abstract

Intraoperative cancer diagnosis using Raman spectroscopy is a growing area of research, but clinical adoption is held back by relatively slow single point data acquisition. Its application to glioblastoma is particularly attractive because of the distinct spectra of malignant and healthy brain tissues, which allow detection of even diffusely invasive glioblastoma with high accuracy. Current clinical practice for intraoperative guidance uses indocyanine green (ICG) for wide-field localization, but its nonspecific accumulation beyond tumor margins leads to false-positive guidance. Critically, the strong fluorescence background from ICG under 785-nm Raman excitation overwhelms the inherently weak Raman signal, thereby preventing concurrent use of the 2 modalities. Here, we overcome this barrier by introducing a signal-processing algorithm that suppresses ICG-derived interference, enabling coregistered acquisition. We present an integrated “search-and-confirm” workflow, where ICG first identifies regions of interest and subsequent signal processing suppresses fluorescence interference, thereby enabling machine-learning-based Raman diagnosis within 3 s and achieving 85% to 90% identification accuracy in vivo. By enabling spatial correlation, we demonstrate that ICG systematically overestimates tumor extent, while Raman signatures match histopathological margins, thereby transforming Raman spectroscopy into a real-time corrective filter for clinical fluorescence guidance and offering a practical path toward precision glioblastoma resection.

## Introduction

Glioblastoma is the most aggressive type of primary brain tumors in adults, and surgical resection remains a cornerstone of therapy [1,2]. However, identifying the exact boundary between invasive tumor cells and normal brain tissue during surgery is a major challenge because glioblastoma cells infiltrate beyond the contrast-enhancing lesions [3]. In response, fluorescence-guided surgery (FGS) has been developed to augment the surgeon’s vision. For example, administration of 5-aminolevulinic acid (5-ALA) leads to accumulation of protoporphyrin IX in glioma cells, producing a pink fluorescence under blue light [4,5]. Similarly, near-infrared (NIR) fluorophores such as indocyanine green (ICG) have been used for fluorescence visualization and image-guided resection [6,7]. While contrast-enhanced fluorescence imaging is well suited for finding suspicious lesions at high sensitivity across a large surgical field, the fluorescence signals are not tumor specific and often lead to false-positive detection [8,9]. For example, ICG accumulates wherever the blood–brain barrier is disrupted, which can include peritumoral edema or inflammation [10]. As a result, surgeons cannot solely trust fluorescence, and frozen tissue pathology is often used for validation.

Here, we report the integration of wide-field fluorescence imaging and label-free Raman spectroscopy for in situ identification of brain tumors during surgery. Each tissue type yields distinct Raman spectral signatures (a series of peaks corresponding to biomolecular vibrations), allowing nondestructive “optical biopsy” or fingerprinting of normal and cancerous tissues [11–14]. In glioblastomas, Raman spectral differences arise from variations in the protein, lipid, and nucleic acid contents, for example, tumor regions often show elevated nucleic acid and reduced lipid signals relative to normal brain [15,16]. Importantly, Raman spectroscopy does not require any external or exogenous labels; the contrast comes from endogenous molecules. Prior studies have demonstrated the power of Raman “optical fingerprinting” in neurosurgery. A handheld fiber-optic Raman probe applied to glioblastoma patients’ brains was able to detect invasive tumor (>15% cancer cell infiltration) with about 93% sensitivity and 91% specificity in vivo [17]. The Raman spectral differences between normal brain and glioma tissue are sufficiently diagnostic that machine learning classifiers can achieve around 90% to 95% accuracy in real time [18–20] and have led to clinical trials of Raman-only approaches to in vivo diagnosis [21,22]. These successes illustrate how Raman spectroscopy can serve as a rapid intraoperative pathology tool. However, its fundamental limitation remains its point-by-point acquisition scheme, which is prohibitively slow for surveying a large surgical field. This creates a critical technological gap: We possess a tool for molecular confirmation but lack an efficient strategy to guide its use to the most diagnostically ambiguous locations.

The complementary nature of fluorescence (sensitive, wide-field “search”) and Raman (specific, pinpoint “confirmation”) makes their integration a logical pursuit. However, a truly synergistic combination is nontrivial. The strong fluorescence background from exogenous ICG under laser excitation can easily overwhelm the inherently weak Raman signals, making concurrent data acquisition and interpretation a substantial technical hurdle [23]. To this end, we first established a complete technical pipeline encompassing optimized ICG dosing, defined Raman acquisition parameters, and a robust fluorescence rejection algorithm, thereby decisively overcoming this interference challenge. Having secured robust signal acquisition, we then demonstrate an integrated imaging platform for in vivo applications that synergizes wide-field ICG-induced fluorescence with pinpoint Raman spectroscopy. Unlike previous work utilizing endogenous fluorescence or surface-enhanced Raman scattering (SERS) techniques requiring nanoparticle intervention [24–26], our method leverages a Food-and-Drug-Administration-approved ICG without the need for nanosensors, facilitating clinical translation and enabling real-time surgical guidance with molecular verification. To validate this integration, we used signal-to-noise ratio (SNR) thresholds of ICG fluorescence, previously correlated with tissue pathology [27], to map brain tissues into distinct regions. Crucially, we then subjected each region to Raman spectroscopy to establish its molecular ground truth. This is essential because fluorescence intensity can be a surrogate marker, but Raman spectra directly interrogate the underlying biochemistry, enabling us to verify whether a fluorescence-positive region truly possesses the molecular signature of glioma and not just a disrupted blood–brain barrier. Analysis of these molecular signature revealed that both the signature (800 to 1,800 cm^−1^) and high-wave-number (HWN; 2,800 to 3,050 cm^−1^) bands were highly meaningful for tissue diagnosis, yielding classification accuracies of 85% to 90%.

Furthermore, spatial correlation analysis reveals that the 2 modalities operate not merely in complement but in synergy. Central to this advance is our resolution of a specific technical conflict: the strong fluorescence background from ICG under 785-nm excitation, which has historically precluded concurrent Raman acquisition. Through a tailored signal processing algorithm, we enabled truly coregistered, simultaneous data acquisition, allowing Raman spectroscopy to act as a real-time corrective filter. By providing molecular-specific validation at each ICG-highlighted location, our approach directly addresses the key clinical limitation of ICG, namely, its nonspecific accumulation beyond true tumor boundaries. In contrast to the destructive and delayed nature of conventional histopathology, this integrated platform enables in situ, nondestructive mapping during surgery. Importantly, this coregistered framework allows the direct spatial comparison and quantitative demonstration that ICG systematically overestimates tumor margins, whereas Raman signatures align accurately with histopathological edges. While further clinical integration remains necessary, this work establishes a technically feasible and pathologically validated paradigm that shifts intraoperative guidance from dual-modal imaging toward an intelligent, self-validating surgical navigation system.

## Results and Discussion

### Machine learning classification of brain tumors and healthy brain tissues

**Fig. 1.**
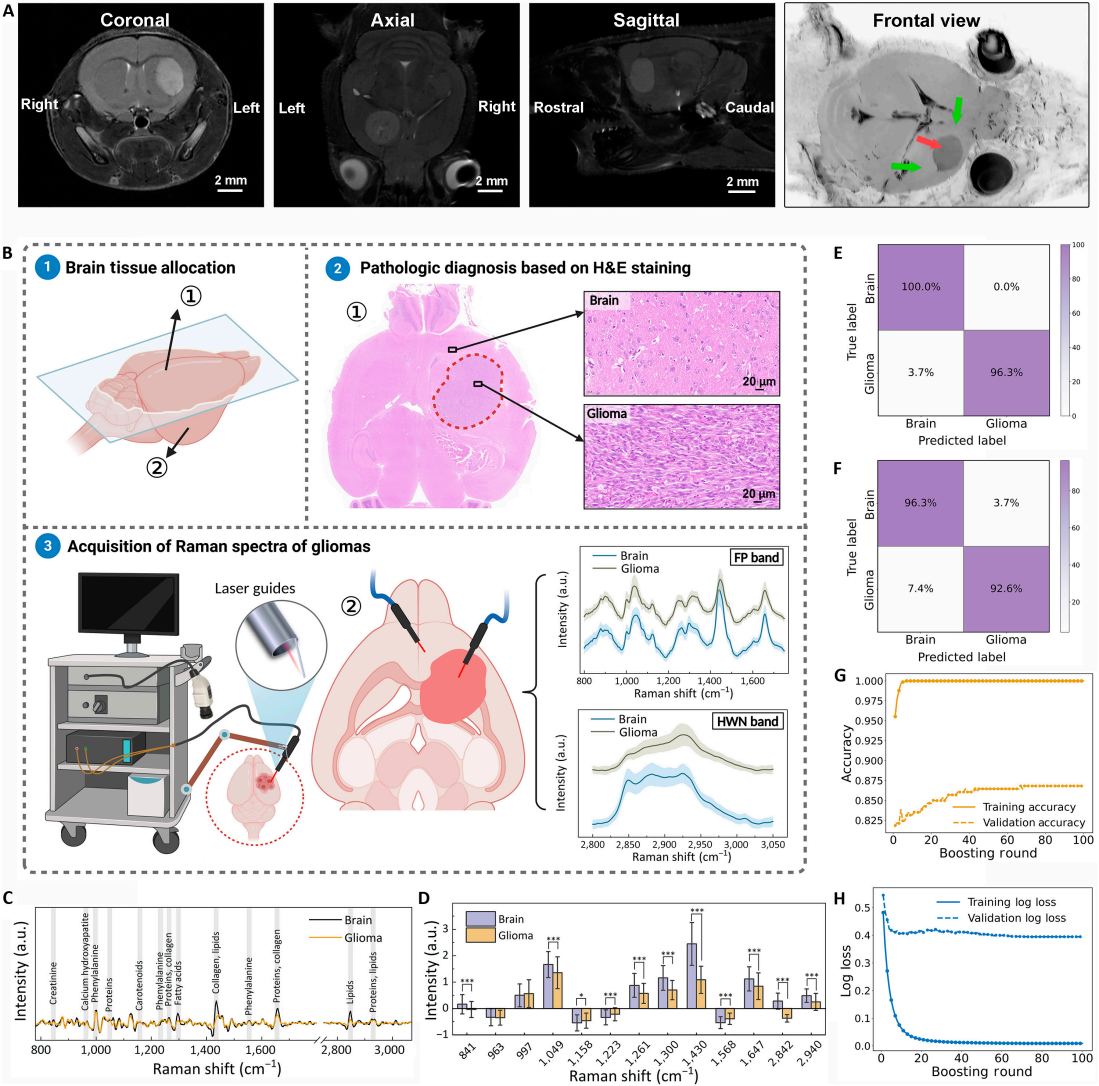
(A) Magnetic resonance imaging (MRI) of mouse brain (coronal, axial, sagittal views; scale bars, 2 mm) highlighting glioma (red arrow) and brain tissue (green arrows). (B) Pathological validation of Raman spectroscopy for distinguishing glioma from brain tissue. Solid lines denote mean spectra; shading represents standard deviations. (C) First-order derivative Raman spectra with characteristic peaks (arrows). (D) Intensity comparison of characteristic peaks between brain and glioma tissues. Significance levels were defined as *P < 0.05 and ***P < 0.001. (E and F) Confusion matrices for Raman fingerprint (FP) and high-wave-number (HWN) bands. (G and H) XGBoost model performance: learning curves and loss metrics.

This study was designed to construct a comprehensive handheld Raman database for glioma and develop a highly accurate classification model to differentiate glioma from healthy brain tissues. To ensure biological relevance and reproducibility, we established a murine glioma model and rigorously validated it using contrast-enhanced T1-weighted magnetic resonance imaging (MRI). High-resolution visualization was achieved through multidimensional reconstructions across coronal, axial, and sagittal planes, facilitated by RadiAnt DICOM Viewer software (Fig. 1A). The handheld Raman probe’s excitation spot, limited to hundreds of microns, poses challenges in aligning with micron-scale pathological regions, a common issue for in vivo Raman diagnosis [12,28,29]. To minimize spatial misalignment, we used a hemispheric comparison method, with one brain hemisphere bisected for hematoxylin and eosin (H&E) staining to confirm glioma pathology (Fig. 1B, upper portion). Tumor regions were outlined with red dashed lines, and inset magnified images were included to detail structural features, providing a clear visualization of the glioma boundaries and pathological characteristics.

Following this pathological validation, the contralateral hemisphere was allocated to Raman spectroscopy, aiming to distinguish glioma from healthy brain tissues at the molecular level, thereby complementing pathological findings with molecular insights. Using a 785-nm excitation wavelength, orthogonal experiments (detailed in Supplementary Materials, Fig. S1, and Tables S1 to S3) were conducted to optimize acquisition parameters, with the SNR value of characteristic Raman peaks serving as the primary evaluation criterion. This systematic approach identified the optimal combination as 3-s exposure time, 250-mW laser power, and single accumulation. Accurate spectral acquisition was ensured by stabilizing the Raman probe with a robotic arm, while the laser guide at the probe’s tip facilitated precise targeting and optimal laser contact, minimizing probe vibration and enhancing data stability (Figs. S2 and S3). The laser guide also maintains a fixed working distance of 3 mm between the probe tip and tissue surface, corresponding to the optimal focal position as determined by SNR comparisons across varying distances (Fig. S4). Assessment of the mean standard deviation in biological tissue across varying laser powers revealed a substantial decrease from 329.07 ± 266.12 under manual operation to 26.06 ± 13.65 under guided operation for the peak at 1,442 cm^−1^ and from 131.87 ± 111.88 to 13.73 ± 2.42 for the peak at 1,654 cm^−1^ (Fig. S5). These reductions correspond to an approximately 12- and 9.6-fold improvement in spectral stability for peaks at 1,442 and 1,654 cm^−1^, respectively. Furthermore, intermittent laser excitation was used to ensure high-quality spectral acquisition while effectively mitigating thermal effects. As shown in Fig. S6, experimental measurements confirmed that continuous irradiation under room temperature produced only a modest temperature (~1 °C) rise in tissue, which remains well below the threshold for thermal tissue damage.

A minimum of 50 spectra were collected per sample, with pathologists monitoring data acquisition to ensure consistency with pathological findings. Subsequent spectral preprocessing was performed, as detailed in the Supplementary Materials and Fig. S7. The Raman spectral data were categorized into 2 key regions (Fig. 1B, lower portion): the FP band, rich in proteins, lipids, and nucleic acids, and the HWN band, containing fewer signals from proteins and lipids [30,31]. Marked spectral differences were observed between brain and glioma tissues, with diagnostically relevant Raman peaks identified (Table S4). First-order derivative spectra with intensity plots highlighted these differences (Fig. 1C and D). Notably, peaks at 963, 1,261, 1,430, and 1,647 cm^−1^ were markedly higher in brain tissue. These peaks are associated with structural proteins (amide I), lipids, and amino acids, consistent with the dense [32], organized microstructure of normal neuropil. In contrast, their reduced intensity in glioma tissue suggests underlying disruptions in lipid content and protein architecture, which are hallmarks of malignant transformation. Conversely, glioma showed an elevated signal around 997 cm^−1^, a peak frequently associated with phenylalanine, whose accumulation is linked to increased protein synthesis in proliferating tumor cells. In the HWN band, Raman spectroscopy also effectively discriminates glioma from normal brain tissue. The characteristic peaks in this range arise primarily from CH_2_/CH_3_ stretching vibrations of lipids and proteins, with glioma exhibiting enhanced signal around 2,940 cm^−1^, consistent with its elevated protein metabolism, whereas normal brain tissue shows more prominent lipid-associated bands (2,842 cm^−1^), reflecting its myelin-rich structure. Collectively, these spectral features not only distinguish glioma from normal brain but also mirror underlying metabolic and structural dysregulation, thereby providing a biological rationale for precision resection.

Subsequently, the XGBoost algorithm was used for its superior performance and computational efficiency in handling complex, large-scale datasets [33,34]. Performance metrics, including accuracy, sensitivity, and specificity, were calculated for each validation, with confusion matrix analyses providing detailed insights into the model’s classification performance. For the FP band, the model achieved mean sensitivity, specificity, and accuracy of 88.3%, 85.3%, and 86.8%, respectively (Fig. 1E). For the HWN band, corresponding values were 86.4%, 84.7%, and 85.6% (Fig. 1F). Following ~20 boosting rounds, both accuracy and log loss reached steady states (Fig. 1G and H), demonstrating robust generalization to the independent validation set, where classification accuracy exceeded 85 %.

### Optimization of ICG concentration and fluorescence removal

The concurrent use of fluorescence imaging and Raman spectroscopy for intraoperative tumor navigation is challenged by the strong ICG fluorescence background under 785-nm laser excitation, which substantially interferes with Raman signal detection [23,35]. To establish a concentration range that balances fluorescence contrast with Raman signal integrity, we systematically expanded upon our preliminary work by evaluating ICG concentrations from 10^−10^ to 10^−7^ M [36]. Through fluorescence imaging (power density, 168.4 mW/cm^2^; integration time, 50 ms; total acquisition, 20 s) combined with Canny edge detection, the optimal concentration window was determined to be 10^−10^ to 10^−9^ M. This range achieves clear tumor boundary delineation (Fig. S8) while minimizing interference with Raman signal acquisition. The fitting and temporal evolution of the fluorescence peak under continuous laser excitation are provided in Fig. S9.

**Fig. 2.**
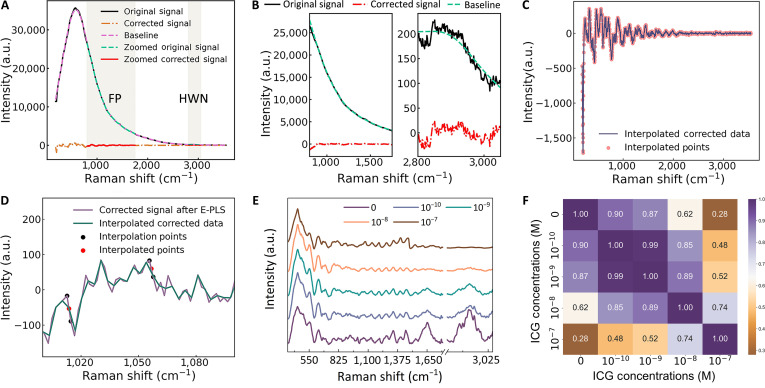
Baseline correction and separation of Raman and fluorescence signals using the extended partial least squares (E-PLS) method and linear interpolation. (A) Comparison of original and E-PLS corrected signals. (B) Magnified fingerprint (FP) and high-wave-number (HWN) Raman signals in the original and E-PLS corrected data. (C) Raman spectra of pork samples labeled with different indocyanine green (ICG) concentrations after data processing. (D) Magnified view of corrected signal after linear interpolation. (E) Interpolated corrected data with interpolation points. (F) Pearson correlation coefficients of preprocessed Raman signals.

The extraction of Raman spectral features for quantitative analysis is fundamentally constrained by the pervasive fluorescence background, which obscures the inherently weak inelastic scattering signals. To resolve this, we used an advanced spectral decomposition strategy that integrates extended partial least squares (E-PLS) with linear interpolation [37,38]. This approach transcends conventional baseline fitting by leveraging the distinct statistical natures of the Raman signal and the fluorescence background. The E-PLS algorithm formulates the baseline estimation as a regularized optimization problem, mathematically expressed as follows:

$$\text{Baseline}=w+\lambda D^{T}D$$ (1)

w constitutes the projection matrix derived from the latent variables of the fluorescence component, λ serves as the regularization parameter that governs the trade-off between baseline fidelity and smoothness, and D is the finite-difference matrix whose transpose $D^{T}$ enforces the smoothness constraint on the solution. The core of the method lies in its ability to iteratively isolate the 2 components based on their covariance structures, rather than relying on arbitrary morphological fitting. The final, corrected Raman signal is subsequently obtained by subtracting this physically plausible baseline estimate from the original measured signal. Figure 2A demonstrates the efficacy of E-PLS in removing fluorescence interference, with Fig. 2B highlighting the improved clarity in critical FP and HWN bands.

In high-fluorescence scenarios, the residual signal after E-PLS subtraction can occasionally exhibit minor artifacts or discontinuities, particularly in regions of steeply varying fluorescence background. To ensure spectral continuity for subsequent machine learning analysis, we applied a local linear interpolation as a postprocessing step. This method was chosen for its simplicity and its ability to preserve local spectral gradients without introducing the spurious oscillations typical of higher-order interpolants. It is mathematically expressed as follows:

$$y=y_{1}+\frac{\;\left(x-x₁\right)\left(y₂-y₁\right)}{x₂-x₁}$$ (2)

where the coordinates (x₁, y₁) and (x₂, y₂) represent the intensities of 2 known, contiguous data points flanking the gap and y denotes the interpolated intensity at the desired position x. By systematically applying this rule across discontinuities, the algorithm performs a piecewise linear reconstruction of the signal. This approach not only restores signal continuity but, more importantly, also preserves local spectral gradients while avoiding the spurious oscillations that may arise from higher-order interpolation. As demonstrated in Fig. 2C and D, the proposed preprocessing strategy offers a simple yet effective solution for Raman analysis in high-fluorescence environments, requiring no additional hardware such as dual-wavelength lasers or supplementary optical filters [39,40].

Pearson correlation analysis (Fig. 2E and F) quantitatively defines the operational window for dual-modal imaging. The high spectral fidelity (87.0% to 90.0%) maintained at ICG concentrations of 10^−10^ to 10^−9^ M confirms that molecular-specific Raman diagnosis remains viable within this range. The subsequent sharp degradation in correlation (28% to 62%) at higher concentrations clearly delineates the boundary beyond which fluorescence overwhelms the Raman signal, underscoring the critical importance of precise dose control. By defining the optimal ICG concentration and implementing a dedicated spectral processing pipeline, this work successfully mitigates the fluorescence–Raman interference, thereby enabling dual-modal imaging and paving the way for precise intraoperative tissue characterization.

### Ex vivo validation of fluorescence–Raman integration

**Fig. 3.**
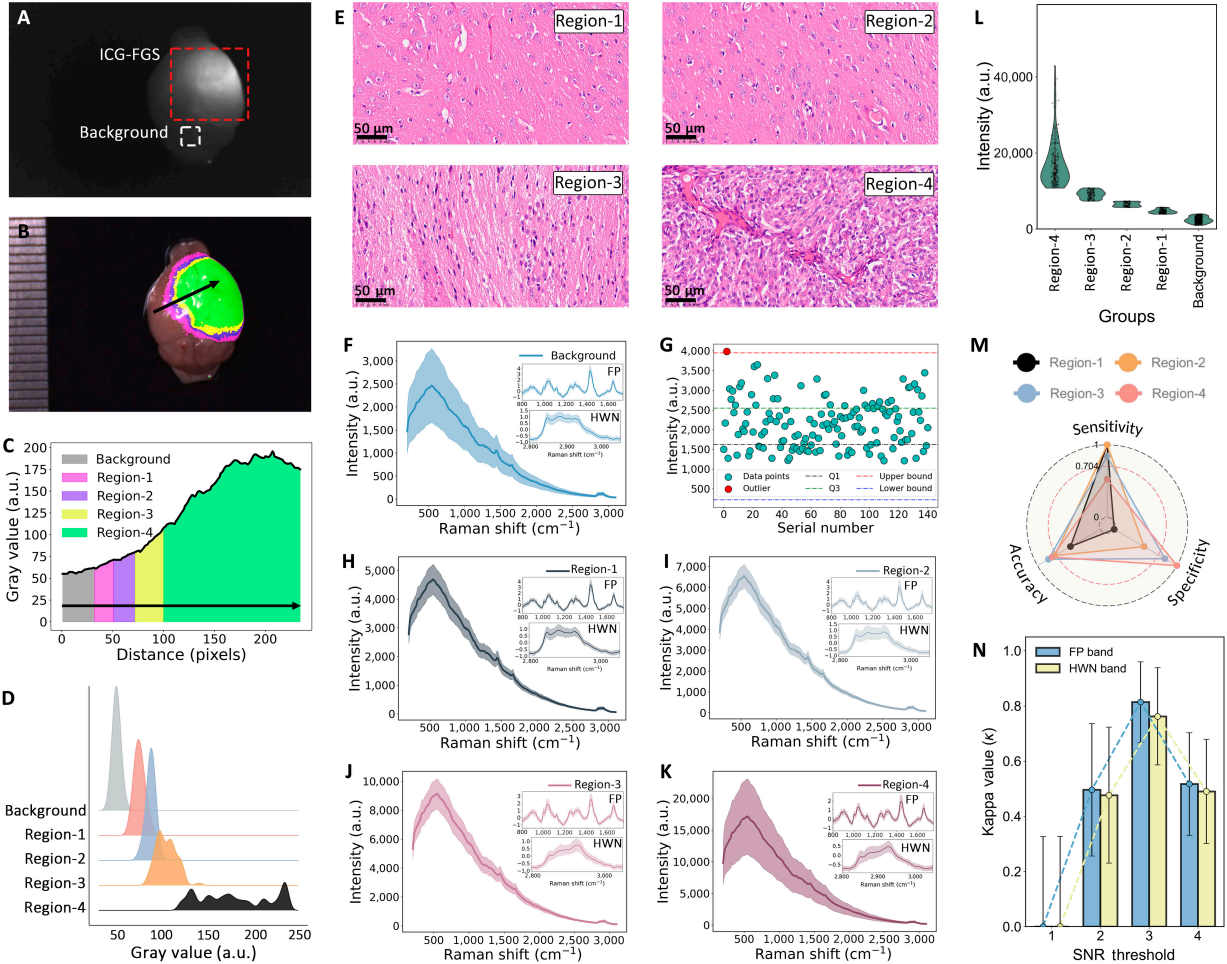
Signal-to-noise ratio (SNR) mapping of brain tissues into distinct regions, and corresponding histopathological and Raman spectral data. (A) Indocyanine green (ICG)–fluorescence-guided surgery (FGS) fluorescence grayscale image of brain tissue. (B) Four fluorescence regions with varying intensities. (C) Gray value curve along the indicated path, segmenting fluorescence zones (region-1 to region-4). (D) Gray value distribution for background and fluorescence regions. (E) Hematoxylin and eosin (H&E)-stained histology corresponding to fluorescence regions. (F) Full Raman spectrum of tissue background and preprocessed spectra. (G) Brain autofluorescence intensity distribution. (H to K) Full Raman spectra and preprocessed spectra of fluorescence regions (region-1 to region-4). (L) Fluorescence intensity comparison at 531 cm^−1^. (M) Radar chart of diagnostic metrics for fluorescence labeling validated against pathology. (N) Cohen’s kappa (*κ*) analysis for different SNR thresholds between fingerprint (FP) and high-wave-number (HWN) bands.

Building upon the established correlation between tumor and surrounding tissue fluorescence variance for precise margin delineation, glioma-bearing mice were intravenously administered ICG (2 mg/kg) to achieve intralesional concentrations within the optimized range of 10^−10^ to 10^−9^ M [36]. At 24 h postinjection, the mice were euthanized, and their brains were harvested for ex vivo surface scanning under a fluorescence navigation system equipped with a 785-nm laser excitation source. Grayscale fluorescence images (Fig. 3A) were acquired to visualize regions of elevated ICG accumulation relative to the background. The integrated software of the imaging system performed fluorescence segmentation by designating tissue autofluorescence as the baseline and implementing predefined intensity thresholds (Fig. 3B). Critical to this process was the computation of the SNR, a robust metric for quantifying fluorescence specificity, according to the equation:

$${\mathrm{SNR}}_{i}=\frac{S_{i}-\mu_{B}}{\sigma_{B}}$$ (3)

where $S_{i}$ is the tumor signal intensity at each point, $\mu_{B}$ is the mean reference signal of healthy tissue, and $\sigma_{B}$ is its standard deviation. This approach extends our prior validation by encoding SNR values using a pseudo-color scheme [27], categorizing fluorescence into 4 progressively intensifying regions: background (SNR = 0), region-1 (SNR = 1), region-2 (SNR = 2), region-3 (SNR = 3), and region-4 (SNR ≥ 4). This standardized framework enhances the reproducibility and precision of tumor margin identification, minimizing user-dependent variability and addressing the limitations of traditional labeling methods in complex scenarios. The gray value profile along the arrowed path (Fig. 3C), derived from fluorescence segmentation (Fig. 3B), was analyzed to examine fluorescence distribution and its histological correlation. The fluorescence intensity curve identified 4 distinct regions (region-1 to region-4) based on increasing intensity thresholds.

Figure 3D illustrates distinct fluorescence intensity peaks across these regions, with increasing gray values aligning with the defined thresholds. Histological examination (Fig. 3E) confirmed the segmentation: Region-1 displayed normal brain tissue; region-2 and region-3 exhibited mild cell proliferation, vascularization, and varying tumor infiltration; and region-4 reflected dense tumor cellularity with pronounced morphological abnormalities. Notably, region-1 was histologically confirmed as normal tissue, yet it exhibited fluorescence intensity above the segmentation threshold (SNR = 1). This exemplifies the false-positive dilemma of fluorescence-based segmentation, where nonspecific ICG accumulation in intact but compromised tissue can lead to the misclassification of normal tissue as suspicious.

In this study, fluorescent regions were identified using system software, with 10 to 20 Raman spectra acquired per region, each collected in 3 s. Figure 3F presents the Raman spectrum of the tissue background, serving as a reference for evaluating the influence of fluorescence-segmented regions on Raman signal interpretation; raw data were preprocessed for display. The baseline for tissue autofluorescence intensity was established by analyzing spectra from ICG-free brain tissues, using an interquartile-range-based outlier detection method to define a safe threshold. As shown in Fig. 3G, green dots represent points within the normal range, while red dots indicate outliers, leading to a critical autofluorescence value of 3,941.62. Figure 3H to K displays Raman spectra from fluorescence-segmented regions (region-1 to region-4), with intensity profiles progressively increasing in higher fluorescence intensity regions. Insets in each panel provide magnified views of the FP and HWN bands, revealing molecular-level differences across fluorescence regions.

Fluorescence peak intensities above the autofluorescence threshold were similarly observed in region-1, indicating the presence of false-positive signals. Fluorescence background intensities extracted from raw Raman spectra (Fig. 3L) show a pronounced substantial gradient between tumor and normal regions, mirroring fluorescence-based segmentation and supporting the precision of boundary delineation. To evaluate the feasibility of the dual-modality technique, this study compared the diagnostic results of fluorescence alone and the fluorescence–Raman technique based on pathological findings. The comparison between the ICG-defined fluorescence regions and histological findings, visualized through radar charts (Fig. 3M), shows region-3 demonstrating superior performance across all directions (82.5% accuracy), similar to previously reported results [27].

Given that Raman spectra collected from different fluorescence regions represent a mixture of brain and glioma tissues, the preprocessed average spectra exhibit distinct profiles across regions. To delineate the contribution of each tissue type, we used the XGBoost classification model, trained in the previous section, to predict the likelihood of each spectrum originating from brain or glioma. The model sequentially fed unlabeled data samples from different regions into the pretrained XGBoost classifier, calculating the contribution of each feature to the decision trees based on learned weights and patterns. Using a predefined decision threshold, samples with a predicted probability exceeding 0.5 were classified as glioma (class 1), while those with a probability of 0.5 or below were assigned to brain (class 0). Raman spectral analysis reveals a complex and informative relationship with fluorescence-based segmentation. The progression from region-1 to region-4 shows a monotonic increase in the Raman-classified tumor probability, which correlates strongly with increasing fluorescence SNR and histologically confirmed tumor burden. This demonstrates that fluorescence segmentation effectively enriches for regions of varying tumor likelihood, which Raman spectroscopy then quantifies with molecular specificity.

The detailed classification results are visualized in Fig. S10 and Tables S5 to S8. When Raman predictions are compared to ICG-defined regional classifications, region-3 exhibited the highest agreement, with 91.2% concordance in the FP band and 85.4% in the HWN band, consistent with this region’s typical histological composition of 60% to 70% tumor infiltration. In contrast, region-1 showed minimal agreement with tumor classification (58.0%), consistent with its histologically validated status as consistently cancer-free despite low-level fluorescence signals. The behavior of the XGBoost classifier in region-2 is particularly insightful. The model’s intermediate predicted probabilities (often near the 0.5 decision threshold) and the resultant ~77% concordance with either pure class do not indicate a failure but rather a successful quantitative capture of the underlying tissue heterogeneity. This probabilistic output provides a continuous measure of tumor infiltration that aligns with the histological finding of approximately 20% tumor cell presence in this transitional zone, a nuance completely lost in the binary output of fluorescence imaging alone.

To directly compare the diagnostic performance of the fluorescence-image-SNR-based segmentation and the Raman approaches, we performed (*κ*) analysis using each SNR threshold as the diagnostic boundary (Fig. 3N). Inter-rater agreement analysis revealed that threshold 3 demonstrated optimal concordance between the 2 methods, with *κ* = 0.814 (95% confidence interval [CI], 0.668 to 0.960) for the FP band and *κ* = 0.762 (95% CI, 0.586 to 0.939) for the HWN band, indicating almost perfect to substantial agreement. Correlation analysis further confirmed this relationship, with perfect Spearman correlations (*ρ* = 1.000, *P* < 0.001) and strong Pearson correlations (*r* > 0.93, *P* < 0.06) between both methods and histological findings (Fig. S11). This variability underscores the spectral heterogeneity within ICG-defined regions, likely driven by the varying degrees of tumor infiltration or disparities in fluorescence intensity due to uneven dye uptake. Glioma cells exhibit infiltrative gradients along vascular and white matter structures, and our multipoint Raman sampling with probabilistic outputs in heterogeneous regions captures these biologically relevant gradients [41,42]. The strong intermethod agreement (*κ* > 0.76 at optimal threshold) demonstrates that fluorescence–Raman integration provides highly correlated molecular information across spatially defined regions, offering robust complementary characterization for in vivo tissue analysis.

### In vivo evaluation of Raman–fluorescence integration

**Fig. 4.**
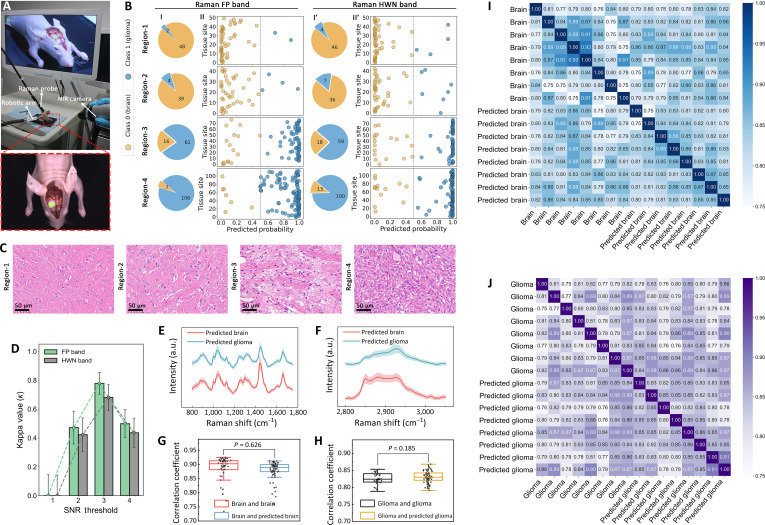
Integrated indocyanine green (ICG) fluorescence and Raman spectroscopy of brain tumors in live animal models showing strong correlation with tissue pathology. (A) Near-infrared (NIR) imaging of ICG–fluorescence-guided surgery (FGS)-marked gliomas using a 785-nm light source. (B) Scatter plots of predicted probabilities for Raman spectra across fluorescence-segmented regions (divided into fingerprint [FP] and high-wave-number [HWN] bands), with pie charts showing brain and glioma distributions. (C) Hematoxylin and eosin (H&E)-stained histology corresponding to fluorescence regions. (D) Cohen’s kappa *(κ)* analysis for different signal-to-noise ratio (SNR) thresholds between FP and HWN bands. (E) Raman spectra of predicted tissues in the FP band. Solid lines represent mean values, and shading indicates standard deviations. (F) Raman spectra of predicted tissues in the HWN band. Solid lines represent mean values, and shading indicates standard deviations. (G) Correlation heatmap between actual and predicted brain tissue. (H) Correlation heatmap between actual and predicted glioma tissue. (I) Comparison of correlation coefficients for brain tissue. (J) Comparison of correlation coefficients for glioma tissue.

Building on in vitro fluorescence–Raman correlations, in vivo experiments were conducted to evaluate Raman spectroscopy for rapid molecular tissue characterization. Figure 4A illustrates fluorescence scanning and Raman spectroscopy for glioma detection in brain tissue 24 h after injecting ICG (2 mg/kg) in a living model. Blood was cleared via saline irrigation and suction immediately after craniotomy and prior to Raman measurements, eliminating hemoglobin interference while preserving tissue integrity for accurate spectral acquisition. The lower panel highlights distinct fluorescence regions, with 10 to 20 spectra acquired per region, each collected within 3 s. Following horizontal brain sectioning, pathological analysis was performed for each fluorescent region. Figure 4B shows the classification outcomes of Raman spectra from fluorescence-segmented regions (region-1 to region-4), with predicted probabilities differentiating brain tissue (class 0) from glioma (class 1) across the FP and HWN bands.

Predictions were generated using the XGBoost model, consistent with ex vivo methodologies, and evaluated under a double-blind protocol to ensure unbiased results. Higher fluorescence intensity regions (3 and 4) contained predominantly glioma-classified spectra, while lower intensity regions (1 and 2) were primarily classified as brain tissue (Fig. 4C). As shown in Tables S9 and 10, diagnostic accuracies in the FP band were 61.1%, 77.4%, 89.7%, and 73.8% for region-1 to region-4, respectively, with similar trends in the HWN band (60.0%, 74.9%, 85.2%, and 70.7%). These quantitative in vivo results exhibit strong concordance with our controlled ex vivo findings (Δaccuracy < 5%), with the minor metric reductions primarily attributable to dynamic intraoperative variables such as tissue motion artifacts. Here, our work achieves 89.7% accuracy (FP band) and 85.2% (HWN band), surpassing fluorescence methods alone (82.5%), while avoiding the tissue damage associated with biopsy. Furthermore, based on the in vitro validation results, we proceeded with the Cohen’s kappa *κ* analysis using each SNR threshold as the diagnostic boundary (Fig. 4D). The analysis revealed that threshold 3 demonstrated the best concordance between the 2 methods, with a *κ* value of 0.778 (95% CI, 0.702 to 0.855) for the FP band and 0.681 (95% CI, 0.592 to 0.770) for the HWN band, which is consistent with the in vitro findings.

This validation confirms that fluorescence segmentation not only guides Raman sampling locations but actively augments molecular-specific tissue characterization, as evidenced by the consistent ex-vivo-to-in-vivo correlation of dual-modal diagnostic patterns. Data points near the 0.5 predicted probability threshold likely represent transitional zones with mixed spectral features, indicative of tumor margins or mild infiltration, as corroborated by pathological analysis that showed minor tumor infiltration in region-2 and mixed tissue characteristics in region-3. Our multipoint Raman sampling approach generates probabilistic outputs within fluorescence-defined regions, validated by H&E staining, to capture infiltrative gradients of tumor heterogeneity. Following XGBoost classification, the average spectrum from each prediction class for the 2 bands is shown in Fig. 4E and F. Pearson correlation analysis between predicted and actual spectra showed strong similarity with no statistically significant differences for both brain (*P* = 0.626) and glioma tissues (*P* = 0.185), as illustrated in Fig. 4G and H. Correlation heatmaps (Fig. 4I and J) confirmed strong within-group spectral consistency, validating the model’s reliability for distinguishing brain and glioma tissues in vivo*.*

These findings demonstrate that Raman spectroscopy, based on fluorescence partitioning, provides reliable molecular characterization in regions with well-defined fluorescence signals (region-3 and region-4), where pathological analysis confirms predominantly cancerous tissue that would typically warrant resection under current clinical practice [43,44]. Conversely, regions with minimal fluorescence (region-1) exhibit molecular signatures consistent with normal brain tissue requiring preservation. Transitional regions (region-2) present mixed molecular signatures that correlate with the heterogeneous pathology observed in these zones. While we demonstrate regional rather than point-by-point analysis, the high accuracy achieved in distinguishing clearly normal (region-1) from clearly cancerous (region-4) tissue, combined with the general correlation between Raman and histopathological diagnosis within transitional areas, suggests that Raman signatures could reliably indicate local tissue status.

Given this diagnostic reliability and the independent validation provided by both FP and HWN spectral bands, a potential conservative surgical practice could leverage this dual-band analysis: Areas showing concordant “clean” Raman signatures across both FP and HWN bands in region-2 may be considered healthy tissue suitable for preservation, while areas showing positive tumor signatures in either or both spectral regions would favor resection. While complete removal of every cancer cell remains impossible, this dual confirmation approach maximizes both tissue preservation and tumor removal confidence, potentially improving patient outcomes when combined with standard adjuvant radiotherapy and chemotherapy. This establishes a practical and intelligent surgical workflow: (a) ICG fluorescence provides a real-time, wide-field “suspicion map” to identify regions requiring verification (SNR ≥ 2); (b) rapid, targeted Raman sampling at these sites then provides molecular confirmation, delivering high diagnostic confidence in core tumor regions (SNR ≥ 3) and critical decision support in ambiguous, infiltrative margins (SNR = 2). This “characterize-then-resect” paradigm promises to enhance surgical precision beyond the capabilities of any single modality alone. As summarized in Table S11, this integrated approach translates the high per-spectrum accuracy of Raman into a practical intraoperative workflow by combining the rapid, wide-field targeting of fluorescence with efficient, molecularly specific confirmation, thereby addressing the key limitations of untargeted Raman-only paradigms in time efficiency and spatial guidance.

### Pathological validation and spatial correlation of ICG fluorescence and Raman spectroscopy

**Fig. 5.**
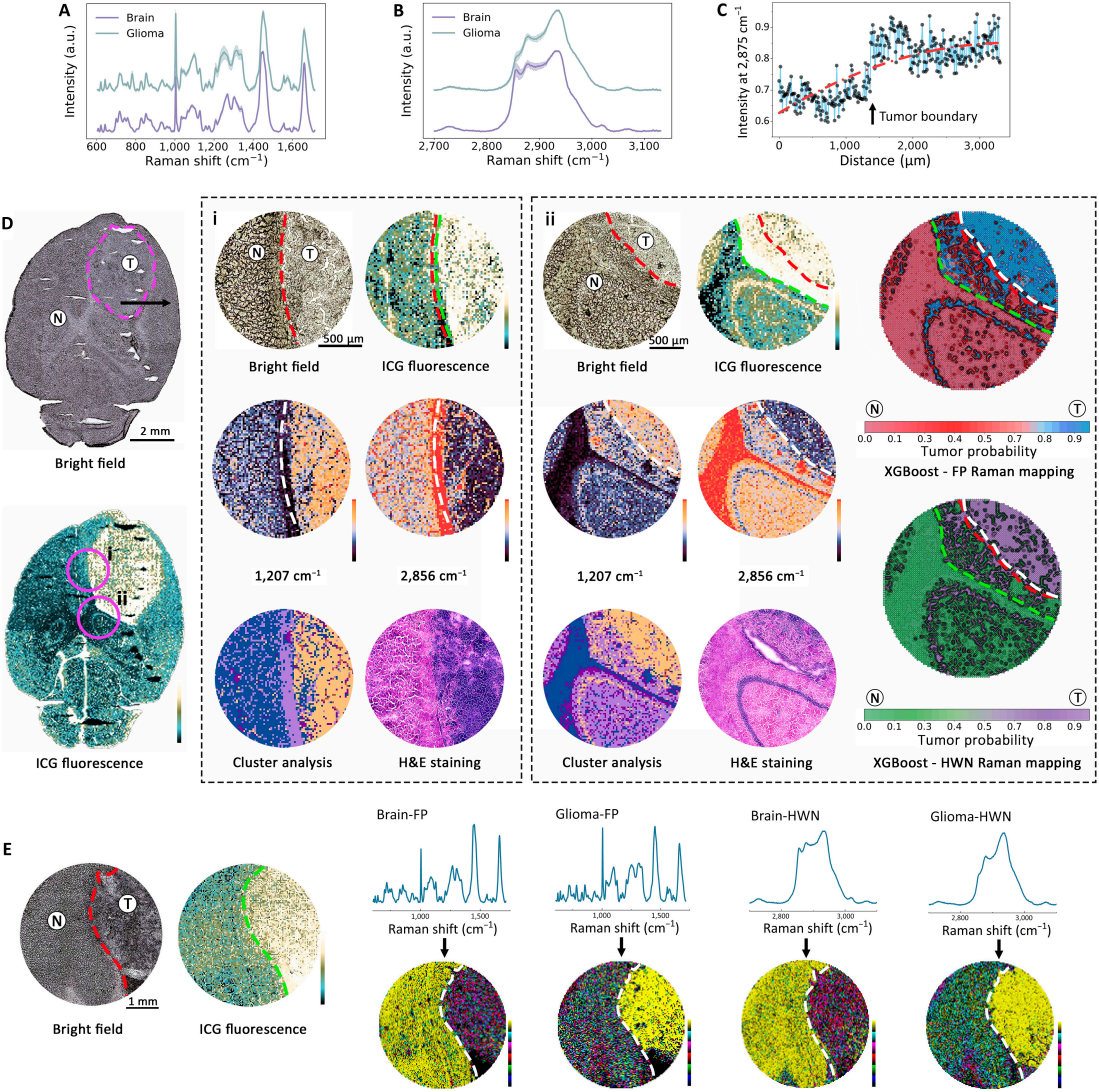
Pathological validation and spatial correlation of indocyanine green (ICG) fluorescence and Raman spectroscopy. (A and B) Raman spectra of classified tissues (fingerprint [FP] and high-wave-number [HWN] bands); solid lines denote mean values, shading represents standard deviations. (C) Variation of the peak intensity at 2,875 cm^−1^ along the black arrow path; red dashed line indicates fitted curve. (D) Raman and ICG fluorescence images of glioma (T) and brain (N) regions; pink dashed lines frame the tumor. Outline highlights magnified areas (i and ii): Raman bright field, fluorescence, spectral maps (FP, 1,207 cm^−1^; HWN, 2,856 cm^−1^), cluster analysis, hematoxylin and eosin (H&E) staining, and contour maps of tumor probability in FP and HWN bands. (E) Raman and fluorescence images with boundaries, spectral profiles, and component analysis maps for brain and glioma tissues in FP and HWN bands. Red dashed line represents the visible tumor boundary, green dashed line represents the fluorescence boundary, and white dashed line represents the Raman boundary.

Spatially resolved Raman mapping of tissue sections provided the most definitive validation of our integrated platform, revealing a fundamental and surgically critical discrepancy between fluorescence guidance and molecular truth. While a clear spatial correlation exists, Raman mapping precisely defined the histopathological tumor boundary, whereas ICG fluorescence consistently overestimated it, demonstrating the unique power of Raman to prevent false-positive resection. In this work, the ICG-injected glioma brain was cryosectioned into 10-μm-thick slices, and Raman spectra were collected from brain and glioma regions using serial pathological sections as reference standards, with mapping performed at a 2-μm step size over approximately 40 min per region of interest. As shown in Fig. 5A and B, the preprocessed Raman spectra exhibited distinct spectral bands compared to those from solid tissues, highlighting differences in spectral profiles after sectioning. This approach builds on earlier band-based classification, exploring whether a single characteristic peak could achieve tissue classification.

Along the trajectory indicated by the black arrow in the Raman image of a whole-brain slice (Fig. 5D), Raman spectra were acquired at 10-μm intervals. The intensity distribution of the peak at 2,880 cm^−1^ revealed a gradient-like increase at the tumor margins, with the fitted curve further corroborating this upward trend (Fig. 5C). To overcome the sensitivity limitations of traditional fluorescence imaging, we acquired fluorescence spectra at the same location on the same tissue slice using confocal Raman fluorescence mode, enabling detailed analysis of characteristic fluorescence peak distributions (Fig. S12). Figure 5D illustrates a comprehensive examination of glioma and brain regional characteristics using ICG fluorescence mapping combined with Raman mapping. Raman mapping revealed an enrichment of proliferative protein-associated signal (1,207 cm^−1^, primarily attributed to tryptophan) in the tumor region, while lipid-associated peaks (2,856 cm^−1^) dominated in the brain tissue (Table S4), reflecting the tumor’s increased tryptophan synthesis due to rapid cell proliferation and reduced lipid content from disrupted myelin structures [45]. In contrast, brain tissue maintained higher lipid levels characteristic of its rich myelin composition [46]. Figure 5D-i shows a detailed comparison of local regions, revealing strong spatial concordance among tumor boundaries delineated by histology (red dashed line), fluorescence imaging (green dashed line), and Raman mapping (white dashed line), enabling accurate characterization of tumor margins and tissue composition. Clustering analysis of Raman spectra further supported this delineation by revealing tissue structures in agreement with histopathological evaluation.

Critically, a detailed comparison of boundaries (Fig. 5D-i and ii) revealed a strong spatial concordance between the tumor margin defined by Raman mapping (white line) and the histopathological ground truth (red line). In contrast, the boundary delineated by ICG fluorescence (green line) consistently extended beyond the pathological margin, highlighting a risk of false positives. This discrepancy illuminates a fundamental biological insight: The “biological field effect” of a disrupted blood–brain barrier, detected by ICG, presents a larger area than the region of actual tumor cell infiltration, precisely defined by Raman. Our integrated system leverages this difference: Fluorescence identifies a zone of pathological risk, while Raman pinpoints the core disease focus, enabling a more intelligent and tissue-preserving resection strategy. Furthermore, by integrating spectral mapping with unsupervised clustering, we successfully reconstructed tissue composition without prior labels, validating the reliability of our molecular assessment against serial pathology.

To quantitatively and continuously visualize tumor infiltration, we trained an additional XGBoost model using the same training parameters as the handheld model, using confocal Raman spectra of core tumor and distant parenchymal tissue as training data. Despite originating from distinct spectral acquisition systems, handheld and confocal Raman devices exhibited strong correlation in feature importance profiles when analyzed by the same XGBoost model. Using a wavelength tolerance of ±3.5 cm^−1^ to account for minor spectral shifts between instruments [47], the overlap ratio (0.636) and cosine similarity (0.779) in the FP band, which increased to 0.818 and 0.901 in the HWN band, indicate a substantial and biologically meaningful consensus in feature importance between the 2 systems. This demonstrates that the diagnostic Raman signatures are robust and not artifacts of a specific device. These correlation metrics, which quantify both the extent of shared important wavelengths (overlap ratio) and the directional consistency of feature importance distributions (cosine similarity), demonstrate that biologically relevant vibrational signatures are robustly prioritized by the model, irrespective of spectral source (Fig. S13). This model was then applied to the tumor margin of animals not included in the training set to output the probability of malignancy for each mapping point. These probabilities are displayed as a heatmap in Fig. 5D-ii. Pathology-defined margins (red lines) showed near-perfect overlap with Raman-defined regions (white lines), whereas fluorescence-defined areas (green lines) extended into normal tissue, underscoring the risk of relying solely on fluorescence for intraoperative guidance (original images shown in Fig. S14).

To evaluate the robustness of spectral classification across diverse samples, we conducted component analysis using unsupervised clustering algorithms, spatially mapping spectra based on their overall similarities across both the FP and HWN bands. The spatial origins of these clusters were mapped to generate the component analysis images shown in Fig. 5E, clearly delineating boundaries between brain and glioma tissues, with segmentation aligning well with fluorescence-based tumor detection. Interindividual variations in cluster distribution likely arise from underlying biochemical differences, such as diverse cellular composition, metabolic states, and microenvironmental heterogeneity, emphasizing the complexity of tissue classification [48]. Expanding upon prior handheld Raman spectral acquisition, slice-level Raman mapping advances the analysis by delivering spatially resolved tissue classification, facilitating the precise demarcation of glioma and brain regions. This integrative approach transitions from point-based spectral analysis to detailed tissue mapping, providing high-resolution visualization of tumor boundaries and nuanced insights into tissue microenvironments.

## Conclusion

Maximal safe resection of glioblastoma is limited by peritumoral inflammation and blood–brain barrier disruption that obscure the boundary between diffuse infiltrating tumor and healthy tissue. This study demonstrates that ICG fluorescent guidance and Raman spectroscopy can be used together in a dual-modal “search-and-confirm” framework to accurately identify healthy and glioblastoma tissues within this complex environment. The 2 modalities are complementary, with each technique directly addressing the major weakness of the other. Fluorescence guidance provides rapid wide-field screening that would be impractically slow with point-by-point Raman sampling, while Raman corrects fluorescence false positives through molecular specificity. Analysis of fluorescence region-1 demonstrates this clearly. Despite showing elevated ICG fluorescence (SNR = 1), histology confirmed this region as normal brain tissue. Raman classification correctly identified over 90% of spectra from region-1 as noncancerous in both the FP and HWN bands, while fluorescence alone would have flagged this tissue for resection.

More critically, microscopy analysis revealed that Raman-defined tumor boundaries matched histopathological margins on adjacent tissue sections, while ICG fluorescence boundaries systematically extended beyond the true tumor in all samples examined. This discrepancy, observed both ex vivo and in vivo, underscores that ICG alone risks resection of functional brain tissue, while Raman pinpoints the molecular margin. Since ICG accumulation is driven by passive targeting to structural abnormalities such as blood–brain barrier disruption, this overestimation is inherent. It is worth noting that 5-ALA-guided fluorescence surgery, although clinically established and proven to improve the extent of resection, is also limited by its dependence on blood–brain barrier integrity and metabolic activity. Moreover, protoporphyrin IX fluorescence often diffuses beyond the contrast-enhancing tumor region, which may compromise precise boundary delineation [49]. Recent comparative studies of novel targeted probes versus 5-ALA have shown that a cysteine–cathepsin-activatable NIR probe, 6QC-ICG, achieves a higher tumor-to-normal tissue fluorescence ratio than 5-ALA in glioma models, indicating superior targeting specificity [50]. Nevertheless, ICG-based probes still rely largely on passive accumulation driven by structural abnormalities and do not fundamentally overcome the issue of fluorescence signal extending beyond the true molecular tumor border. By overcoming the technical barrier of fluorescence interference, our study enabled coregistered analysis of fluorescence and Raman signals from the same tissue sites, with direct correlation to histopathology. This approach directly demonstrates that both conventional ICG and clinically used 5-ALA can overestimate tumor extent due to their sensitivity to structural rather than molecular cues. In contrast, Raman imaging, by directly probing molecular vibrations, provides tumor boundaries that align closely with the histopathological gold standard.

ICG accumulation is driven primarily by passive targeting to structural abnormalities such as blood–brain barrier disruption rather than tumor-specific molecular characteristics, a well-known limitation of FGS [8–10,51–53]. Direct quantitative comparison of ICG-defined boundaries with molecular tumor margins has been prevented by the technical barrier of fluorescence interference overwhelming Raman signals under shared 785-nm excitation [23]. One effective strategy is shifted-excitation Raman difference spectroscopy, which uses 2 slightly offset excitation wavelengths to separate and subtract the fluorescent background, thereby accurately extracting the Raman signal [54]. Similarly, NIR-II fluorescence leverages its lower autofluorescence and reduced scattering in deep tissue to substantially improve the SNR and enhance the analytical accuracy of fluorescent samples [39]. In contrast to those approaches, the preprocessing method developed in this work combines ICG dosing, E-PLS, and linear interpolation to directly suppress strong fluorescence backgrounds without requiring additional excitation sources or complex spectral shift operations. Our direct comparison of fluorescence and molecular boundaries within the same tissue samples quantifies ICG’s systematic overestimation of tumor extent, which arises from its detection of structural abnormalities rather than malignant cell presence. The conceptual surgical workflow enabled by this integration is efficient: ICG fluorescence rapidly surveys the surgical field to highlight regions suspicious for structural alteration, and Raman spectroscopy then provides targeted, molecular confirmation at these sites within seconds. The 85% to 90% in vivo accuracy of machine-learned classification for these Raman spectra is comparable to prior Raman-only studies [17,20], confirming that the dual-modal integration preserves high diagnostic specificity.

The integration of rapid “search” with precise “confirmation” represents an effective paradigm for addressing ambiguous tumor margins, which is driving the diversification of intraoperative molecular imaging technologies. For instance, targeted spray-on fluorescent probes for the tumor microenvironment, such as real-time fibroblast activation protein *α*-targeted sprays, enable rapid, wide-field visualization of tumor margins and, together with machine-learning-assisted segmentation, integrate rapid panoramic assessment with high-precision confirmation within a unified intraoperative workflow [55]. Parallel to these developments, SERS complements these approaches by delivering dramatically amplified Raman signals for ultrasensitive, multiplexed biomarker detection. Recent studies have demonstrated that automated SERS systems can intraoperatively grade high-risk prostate cancer foci by detecting endogenous tissue acidity and prostate-specific antigen activity [56]. Meanwhile, the integration of targeting ligands, SERS, and fluorescence imaging into a single nanoprobe offers a novel approach for in situ discrimination of breast cancer subtypes [57]. Furthermore, SERS enables the sensitive, multiplexed detection of protein-specific exosomes for ovarian cancer diagnostics, while also demonstrating clinical utility in direct, label-free protein analysis for conditions such as proteinuria [58,59]. The present study utilizes label-free spontaneous Raman spectroscopy for direct molecular fingerprinting, prioritizing clinical translatability and avoidance of exogenous agents. Looking forward, substrate-free Raman enhancement strategies such as stacking-induced charge–transfer-enhanced Raman scattering represent an alternative, multiplexed intraoperative detection without relying on conventional metallic nanostructures [60]. These advances complement our strategy of combining wide-field fluorescence with point-specific Raman spectroscopy at the system level, collectively reflecting a trend toward leveraging multimodal optical methods to enhance the specificity and informational depth of intraoperative diagnosis.

Certainly, it should be noted that this study has certain limitations. The current Raman acquisition speed is adequate for point measurements but too slow for comprehensive mapping of large surgical areas without fluorescence guidance. More critically, although the limited cohort and syngeneic murine model constrain direct extrapolation to human disease complexity, the translational rationale is underpinned by conserved, fundamental biomolecular vibrations that yield diagnostic Raman contrasts across species, a principle already validated in human intraoperative studies [17]. To bridge the translational gap, future work will require defined steps including validating ICG dosage and models in ex vivo human tissues, constructing a large-scale human spectral database, and engineering a sterilizable, miniaturized Raman probe compatible with neurosurgical workflows. Furthermore, we acknowledge that diagnostic granularity could be further refined through more sophisticated multimodal integration, such as developing a unified machine learning model that takes both fluorescence texture features and Raman spectral vectors as combined input to predict infiltration probability.

The “search-and-confirm” paradigm demonstrated here establishes a real-time dual-modal roadmap for glioblastoma surgery, providing molecular-specific confirmation to guide maximal safe resection. Its practical implementation is supported by key technical elements: Laser guidance stabilizes Raman probe–tissue contact; classifier training on spectrally diverse data enhances robustness against tissue heterogeneity; and the use of deeply penetrating 785-nm excitation combined with intraoperative field cleaning minimizes interference from surface fluids, which together enable reliable molecular confirmation within a 3-s acquisition. For clinical translation, engineering challenges include probe miniaturization for surgical accessibility, sterilization protocols compatible with sensitive optics, and integration with existing neurosurgical navigation systems. The integrated fluorescence-plus-Raman “search-and-confirm” approach demonstrated here may be applicable to other solid tumors where FGS is used and margin control is critical, such as head and neck, breast, or colorectal cancers [61–63], although optimization of ICG concentration is likely to be indication specific and tissue-specific models will be required. Finally, this study establishes and validates a real-time, “search-and-confirm” dual-modal imaging paradigm, providing a promising intraoperative roadmap with molecular-specific confirmation toward achieving maximal safe resection of glioblastoma.

## Methods

Detailed methodologies are documented in the Supplementary Materials, encompassing the experimental design, the specifications of the Raman–fluorescence imaging system, the implementation of machine learning models, the spectral data processing pipelines, and the statistical validation framework.

## Data Availability

Research data are available from the corresponding author upon reasonable request.
