# Supplementary material for: Machine-Learning-Enabled Raman Spectroscopy Refines Indocyanine Green Fluorescence Boundaries for Precise Glioblastoma Margin Delineation
Source: Research (Wash D C). 2026 Apr 1;9:1216. doi: 10.34133/research.1216 (PMC13039520; doi:10.34133/research.1216)
Supplement: Supplementary 1 — Figs. S1 to S14 Tables S1 to S11 [file research.1216.f1.docx]

**Supporting Information**

**Machine Learning-Enabled Raman Spectroscopy Refines ICG Fluorescence Boundaries for Precise Glioblastoma Margin Delineation**

Dong Han,^1^ Kai Liu,^1^ Lijun Zhu,^2^ Yufei Miao,^1^ Jinglei Zhang,^1^ Ziyang Wang,^1^ Guangming Lu,^2^ Christopher J. Butch,^1, *^ Huiming Cai,^1, 3, *^ Shuming Nie,^4, *^ and Yiqing Wang^1, *^

^1^Department of Biomedical Engineering, College of Engineering and Applied Sciences, State Key Laboratory of Analytical Chemistry for Life Science, Nanjing University, Nanjing, Jiangsu 210023, China. ^2^Department of Medical Imaging, Jinling Hospital, School of Medicine, Nanjing University, Nanjing, Jiangsu 210002, China. ^3^Nanjing Nuoyuan Medical Devices Co., Ltd, Nanjing, Jiangsu 211500, China. ^4^Department of Biomedical Engineering, University of Illinois at Urbana-Champaign, Urbana, IL 61801, USA.

*Address correspondence to: chrisbutch@nju.edu.cn; caihuiming@nuoyuanmedical.cn; [nies@illinois.edu](mailto:nies@illinois.edu); wangyiqing@nju.edu.cn.

**Figure S1** (a) Range of different factors in orthogonal experiment; (b) Comparison of range values obtained at parameter levels of different factors.

**Figure S2** (a) Illustration of the 3D-printed Raman laser guides model, designed to be securely snapped onto the Raman probe for use; (b) Localized enlargement of the 0.5mm laser guides, red squares with 1mm sides; (c) Schematic diagrams used by the operator at different angles of rotation; (d) Visualization of the effect observed when the laser is activated.

**Figure S3** The surgical imaging system equipment and magnified image of the Raman probe acquisition site.

**Figure S4** Spectroscopic characterization of fatty meat at different focusing distances. (a) Raman spectra recorded at focusing distances ranging from 1 to 10 mm; (b) Comparative histogram illustrating the spectral SNR values of spectra obtained at each focusing distance.

**Figure S5** Raman analysis of fatty tissue using a handheld Raman probe. (a) Representative Raman spectrum of fatty tissue. (b, c) Intensity distributions of the characteristic Raman peak at 1442 cm⁻¹ acquired with 3 s integration at different laser powers: (b) manual handheld operation; (c) measurement with laser guides. (d, e) Corresponding intensity distributions for the peak at 1654 cm⁻¹: (d) manual handheld operation; (e) measurement with laser guides.

**Figure S6** Curves of temperature-time variation of 250mw laser excitation on different tissues.

**Figure S7** Detailed overview of the protocol with Raman analysis.

**Figure S8** (a) Imaging of pork with different concentrations of ICG; (b) Canny edge detection of tissue fluorescence images with gray value variation along the line; (c) Original Raman spectra of pork labeled with different concentrations of ICG.

**Figure S9** (a) plot of major Raman features peaks with increasing ICG concentration; (b) Fitting of the fluorescence peak at 531 cm^-1^ of the raw spectrum; (c) The value of the 531 cm^-1^ peak fluorescence over time using continuous laser excitation at the same point. The solid line is the mean value and the shading is the standard deviation.

**Figure S10** Pathological results statistics of each group, with tumor tissue in the black box and a scale bar of 100 μm.

**Figure S11** (a) Classification rate analysis for FP and HWN bands across different SNR regions; (b) Comparison of histologically positive rate and classification rate for FP and HWN bands.

**Figure S12** Fluorescence spectra of fluorescent negatives and positives measured in the fluorescence mode of confocal Raman: (a) fluorescent negative, (b) fluorescent positive.

**Figure S13** Consistent identification of key spectral features in FP and HWN regions across handheld and confocal Raman platforms. (a, e) Difference spectra with important features identified by XGBoost for handheld and confocal Raman; (b, f) Feature presence distribution with ±3.5 cm⁻¹ tolerance showing overlap (green) between modalities; (c, g) Distribution of important spectral features across wavelength range with matched features highlighted in green; (d, h) Correlation plot of matched features between platforms showing strong linear relationship within ±3.5 cm^-1^ tolerance band.

**Figure S14** Representative images of the original Raman spectral data, bright-field microscopy, ICG fluorescence, H&E staining, and corresponding tumor probability contour maps in the Raman FP and HWN regions.

**Table S1** Parameters of 3-factor 3-level orthogonal experiment.

**Table S2** Table of orthogonal tests.

**Table S3** SNR optimal level screening.

**Table S4**Common Raman band assignments used in cancer diagnostic studies.

**Table S5**The confusion matrix of fluorescent regions with pathology for different thresholds.

**Table S6** iagnostic indices of fluorescence region compared to pathology at different thresholds in Ex Vivo.

**Table S7** he confusion matrix of fluorescent regions with Raman band prediction classification results for different thresholds in Ex Vivo experiments.

**Table S8** Diagnostic indices of gliomas by fluorescent regions and Raman bands at different thresholds in Ex Vivo.

**Table S9** The confusion matrix of fluorescent regions with Raman band prediction classification results for different thresholds in In Vivo experiments.

**Table S10** Diagnostic indices of gliomas by fluorescent regions and Raman bands at different thresholds in In Vivo.

**Table S11** Performance comparison between a Raman-only paradigm and this work.

**Key Resources Table**

| REAGENT or RESOURCE | SOURCE | IDENTIFIER |
| --- | --- | --- |
| Chemicals, peptides, and recombinant proteins | | |
| ICG | Nanjing Nuoyuan Medical Co., Ltd | Custom supply; Registered in China |
| Dulbecco's Modified Eagle Medium | Gibco | Cat No.:11965092 |
| Fetal Bovine Serum | Gibco | Cat No.:10099141 |
| Penicillin-Streptomycin | Gibco | Cat No.:15140122 |
| Trypsin-EDTA | Gibco | Cat No.:25200072 |
| 1X Phosphate Buffered Saline | Gibco | Cat No.:10010023 |
| Experimental models: Cell lines | | |
| Human glioblastoma cell line | ATCC | HTB-14 |
| Experimental models: Organisms/strains | | |
| BALB/c Nude, female, 5-6 weeks | Gempharmatech Co., Ltd | Strain NO. D000521 |
| Software and algorithms | | |
| Surgical Fluorescence Imaging System Software | Nanjing Nuoyuan Medical Co., Ltd | Custom software; Version FLI-10D |
| REAGENT or RESOURCE | SOURCE | IDENTIFIER |
| Python (version 3.9.13) | Python Software Foundation | https://www.python.org |
| XGBoost (version 2.0.2) | Python Software Foundation | https://www.python.org |
| REAGENT or RESOURCE | SOURCE | IDENTIFIER |
| Scikit-learn (version 1.6.1) | Python Software Foundation | https://www.python.org |
| Matplotlib (version 3.5.2) | Python Software Foundation | https://www.python.org |
| Numpy (version 1.23.5) | Python Software Foundation | https://www.python.org |
| WiRE (Version 5.5) | Renishaw | www.renishaw.com/wire |
| Other | | |
| Surgical Imaging System | Nanjing Nuoyuan Medical Co., Ltd | Custom system; Version FLI-10D |
| Technical Specifications of the Surgical Imaging System | \| Excitation wavelength: 785 nm ± 5 nm \| \| --- \| \| Fluorescence spot area: ≥ 20 cm² \| \| Camera resolution: 2048 × 1536 pixels \| \| Compatible Raman spectral range: 170 – 3200 cm⁻¹ \| \| SNR for diagnostic peaks: ≥ 3 \| \| Maximum laser output window temperature: ≤ 60 °C \| | |
| Confocal Micro-Raman System | Renishaw | inVia |

**Experimental Model and Study Participant Details**

**Cell lines**

Human glioblastoma cells (U87MG) were obtained from the American Type Culture Collection (ATCC). Cells were maintained in Dulbecco's Modified Eagle Medium (DMEM, Gibco) supplemented with 15% heat-inactivated fetal bovine serum (FBS, Gibco) and 1% penicillin-streptomycin (Gibco) at 37°C in a humidified atmosphere containing 5% CO₂. Cells were cultured in 75 cm² vented tissue culture flasks and routinely passaged at 80-90% confluence using 0.25% trypsin-EDTA (Gibco) for detachment. Following trypsinization, cells were washed with sterile 1× phosphate-buffered saline (PBS, Gibco) to neutralize trypsin activity and remove residual enzymes. The cell culture medium was replaced every 48-72 hours. All experiments were performed with cells within passages 5-15 after thawing to maintain phenotypic stability.

**Glioma Mouse models**

Female BALB/c nude mice (5-6 weeks old, 20 ± 2 g) were procured from Gempharmatech Co., Ltd (Nanjing, China). Mice were housed in a specific pathogen-free (SPF) facility under controlled environmental conditions: temperature 22 ± 1°C, humidity 50 ± 10%, with a 12-hour light/dark cycle (lights on at 7:00 AM). Animals were housed in individually ventilated cages (IVC) with autoclaved corncob bedding, provided with autoclaved commercial rodent diet and sterile water ad libitum. All animals were acclimatized for at least 7 days prior to any experimental procedures. All animal procedures were approved by the Animal Ethical and Welfare Committee (AEWC) and the Institutional Animal Care and Use Committee (IACUC) of Nanjing University (Approval No. IACUC-2208002), and conducted in accordance with the institutional guidelines for animal welfare.

For constructing tumor models, U87MG cells in logarithmic growth phase were harvested by trypsinization, washed twice with sterile 1× PBS (Gibco), and resuspended in ice-cold sterile 1× PBS at a concentration of 1×10⁵ cells/μL. Cell viability was confirmed to be >95% using trypan blue exclusion assay prior to injection. Mice were anesthetized with 2% isoflurane in oxygen (1 L/min) and positioned in a digital stereotactic frame (RWD Life Science). Ophthalmic ointment was applied to prevent corneal drying during surgery. After scalp disinfection with alternating betadine and 70% ethanol, a midline incision was made to expose the skull. A burr hole was drilled using a micro-drill (0.9 mm drill bit) at the following coordinates relative to Bregma: anteroposterior (AP) +1.0 mm, mediolateral (ML) -1.8 mm. A 10 μL Hamilton syringe (701N, Hamilton Company) with a 26-gauge needle was lowered to a depth of -3.5 mm (dorsoventral, DV) into the striatum. A total of 1×10⁶ cells in 10 μL PBS were injected at a constant rate of 1 μL/min using a micro-infusion pump (KDS100, KD Scientific). The syringe remained in place for 5 minutes post-injection before slow withdrawal (1 mm/min) to prevent reflux. The scalp was closed with 6-0 absorbable sutures, and animals received subcutaneous injection of buprenorphine (0.05 mg/kg) for postoperative analgesia. Body weight and neurological status were monitored daily post-surgery.

Tumor growth was monitored by weekly MRI scans using a 9.4T small animal MRI system (BioSpec 94/30 USR). Ten days post-inoculation, when tumor volume reached 20 mm³ as calculated by the ellipsoid formula:

$$Volume=\frac{\pi}{6}\times\mathrm{length}\times\mathrm{width}^{2}$$

Animals were allocated into three experimental cohorts using block randomization: (1) five animals for establishing the in vitro spectral database; (2) five animals for ex vivo dual-modal validation; and (3) five animals for in vivo validation. From these cohorts, a subset of three animals was randomly selected for detailed tissue sectioning and Raman mapping analysis. Sample size was determined based on our previous studies using similar models and measurements, with power analysis indicating n = 5 per group.

**Pork tissue model preparation**

Fresh lean pork was obtained from a licensed farmers' market in Qixia District, Nanjing, China, within 2 hours post-slaughter. Tissue was transported on ice and processed immediately. Using aseptic technique, tissue blocks were precisely cut to 0.2 cm³ (5 × 5 × 8 mm) using a surgical scalpel and tissue slicer. Tissue blocks were incubated in ICG solutions (Nuoyuan Co., Ltd, China) across a concentration gradient of 10^-10^ M to 10^-7^ M, prepared by serial dilution in deionized water. Incubation was performed in 15 mL conical tubes with 10 mL solution per tube for 12 hours at 4°C with constant shaking at 100 rpm on an orbital shaker (QYC-A, Benting, China). Following incubation, tissues were washed three times with 10 mL ice-cold deionized water (5 minutes per wash) to remove unbound ICG and placed in 24-well plates on filter paper to remove excess water before measurements.

**Tissue processing and sectioning**

For ex vivo measurements, mice were euthanized by CO₂ asphyxiation followed by cervical dislocation. Brains were rapidly harvested and divided sagittally. The left hemisphere was immediately flash-frozen in optimal cutting temperature (OCT) compound (Sakura Finetek) using liquid nitrogen-cooled isopentane and stored at -80°C for cryosectioning. For Raman mapping analysis, glioma tissues were cryosectioned into 10 μm serial sections at -20°C using a cryostat (MEV, SLEE GmbH) and mounted on pre-cleaned aluminum substrates. Consecutive sections at 50 μm intervals were collected on glass slides for histological staining (H&E) to provide pathological correlation. The right hemisphere was used for fresh tissue measurements within one hour post-harvest.

**Method Details**

## **Orthogonal experiments**

To overcome this challenge, we firstly improve the signal-to-noise ratio (SNR) of the spectral data by means of optimizing the experimental conditions and improving the data acquisition method. SNR serves as widely used measure for evaluating the ability of Raman system to extract information from a noisy signal for subsequent analysis. Orthogonal experiments are a scientific method of experimental design used to assess the effects of multiple factors on the results of an experiment. This method works by selectively arranging experiments so that the most information can be obtained in the fewest number of experiments possible.

***1) Determination of experimental factors and levels.*** The three main factors that were determined to influence the results of the experiment were integration time, laser power, and cumulative frequency. Three levels were set for each factor as shown in the Table S1.

***2) Selection of orthogonal tables***. The selection of orthogonal tables is based on the complexity of the experiment and the availability of resources. Therefore, it is important to select the appropriate orthogonal table based on the number of factors and the level of each factor, and for this experiment, a three-factor, three-level orthogonal table was selected.

***3) Assigning experimental programs.*** According to the arrangement of the orthogonal table, the factors and levels are assigned to each experiment and it is ensured that each combination of levels is reasonably tested.

***4) Conduct experiments.*** Conduct the experiments according to the scheme in the orthogonal table, record the results of each experiment, and use the SNR value of the spectra obtained from each experiment as the evaluation index. The orthogonal table as well as the test results are displayed in Table S2.

***5) Data analysis.*** Calculate the effect of each factor on the experimental results. Analyze the experimental results of different combinations of factor levels and determine the best combination of parameters. Calculation of K-value: for each level of each factor, the average response value (i. e. K-value) is calculated. This value reflects the average performance of the experimental results at that level. Selecting the optimal parameter: comparing the K-values of the different levels of each factor, the level corresponding to the maximum K-value of each factor is considered to be the optimal level for that factor. By combining the optimal levels of each factor, the optimal parameter combination for the whole experiment was determined. Selection of optimal parameter combinations: Based on the analysis of K-values, those combinations of factor levels that optimize the experimental results are selected as the optimal parameters.

***6) Evaluating the importance of factors and optimal parameters.*** Statistical methods such as extreme variance analysis was used to assess the degree of influence of each factor on the experimental results, and the importance of each factor was determined to be Integration time, Cumulative frequency, and Laser power in descending order. Based on the experimental data, the experimental optimal level combinations were determined to be displayed in Table S3.

***7) Validate the experimental results.*** Repeat the experiment under the optimal parameter settings to verify the stability and repeatability of the experimental results. Confirm that the optimal parameter combination does produce optimal experimental results.

**Integrated Intraoperative Fluorescence and Raman Imaging**

To establish a co-registered imaging platform for real-time surgical guidance that enables wide-field fluorescence-based tumor "search" with subsequent confirmatory Raman-based molecular "confirmation" in the same session, we employed a custom-integrated FLI-10D system. This dual-modality approach was designed to directly overcome the inherent limitations of standalone techniques, namely the low specificity of fluorescence and the low survey efficiency of Raman spectroscopy. For all in vivo measurements, the system was positioned adjacent to the surgical field. After a 30-minute laser warm-up period to ensure power stability, the sample was illuminated with the 785 nm laser at a power density of 168.4 mW/cm². Wide-field fluorescence imaging was performed by acquiring a sequence of 400 grayscale frames with a 50 ms integration time over 20 seconds; these frames were averaged in real-time by the system software to generate a high SNR image for automated initial tumor margin delineation. Immediately following fluorescence mapping, Raman spectroscopy was conducted at the identified regions of interest (ROIs).

A handheld Raman probe, sterilized with 75% ethanol prior to use, was positioned perpendicular to the tissue surface and stabilized by a robotic arm to maintain a consistent working distance of 6.00 mm. Raman spectra were then acquired using 785 nm excitation at 250 mW power, with a 3 seconds exposure time and a single accumulation per spectrum. For ex vivo validation on harvested tissues and the pork model, the same integrated system was used, but the probe was mounted on a fixed stage for consistent measurement. Weekly wavelength calibration of the Raman spectrometer was conducted using the characteristic 520.5 cm^-1^ peak of a silicon wafer to ensure spectral accuracy.

**High-Resolution Confocal Raman Mapping for Tissue Validation**

High-resolution confocal Raman mapping was performed using a Renishaw inVia system to obtain molecular maps with high spatial resolution for ex vivo validation of the intraoperative system's findings and for precise correlation with histopathology, serving as the molecular "ground truth." During sample preparation, fresh-frozen brain tissues were cryosectioned at 10 μm thickness and mounted on aluminum substrates, with adjacent sections collected for H&E staining to enable subsequent pathological correlation. For system calibration and data acquisition, the system underwent daily wavelength calibration using the characteristic 520.5 cm^-1^ phonon mode of a silicon wafer with ±1 cm⁻¹ tolerance. All measurements were performed at room temperature using a 50× objective (NA = 0.75). The intrinsic lateral (XY) optical resolution of the micro-Raman system with the 100× objective is approximately 0.35 µm, and the spectral resolution is better than 1 cm⁻¹. For tissue mapping, parameters were set at 0.3 s exposure time per point with a 10 μm step size. Laser power at the sample was maintained at 150 mW to prevent photodamage, verified through consecutive spectral acquisitions at the same spot. Spectral data were acquired and initially processed using WiRE 5.5 software (Renishaw). In data collection, Raman maps were specifically acquired across tissue boundaries identified by parallel H&E sections to capture the transition zone from tumor to normal brain. For quality control, laser power was measured at the sample plane before and after each mapping session, and tissue integrity was monitored by comparing the first and last spectra in each map for signs of laser-induced damage.

**Spectral Data Processing and Analysis**

Comprehensive preprocessing of raw spectral data was essential prior to machine learning analysis, primarily due to the substantial interference from tissue autofluorescence and the broad fluorescence peaks from ICG that can overwhelm the weaker Raman signals. Our systematic preprocessing pipeline addressed these challenges through sequential computational steps: First, cosmic rays and stray peaks were identified and removed using an automated algorithm based on derivative analysis, effectively eliminating these sporadic artifacts. Second, E-PLS baseline correction was implemented with specific parameters (λ=600,000, weight=0.01) to separate the broad fluorescence background from the characteristic Raman peaks. Third, spectral data underwent linear interpolation at 1 cm^-1^ intervals to ensure uniform data point distribution across all spectra, creating a consistent foundation for subsequent analysis. Fourth, standardization was achieved using Standard Normal Variate (SNV) transformation, which normalized each spectrum to correct for signal variations arising from differences in sample concentration or thickness. Finally, the preprocessed spectra were segmented into distinct Raman bands for subsequent classification using machine learning algorithms, enabling precise spectral interpretation and tissue identification.

**Experimental Design and Quality Assurance**

To ensure reliability and objectivity of experimental results, strict experimental design principles were followed. For randomization, the sequence of Raman measurements across different fluorescence intensity regions (Region-1 to Region-4) was randomized for each animal to prevent systematic bias. Blinding was implemented during spectral analysis and machine learning classification, where analysts were kept unaware of the fluorescence region designation and histological correlation of each spectrum until model predictions were finalized. Regarding replicates and sample size, a minimum of 50 spectra per tissue type (normal brain and core tumor) were collected for model training, while 10-20 spectra from spatially distinct points within each fluorescence-defined region were acquired for regional validation to account for tissue heterogeneity.

**QUANTIFICATION AND STATISTICAL ANALYSIS**

The dataset was partitioned following a strict subject-wise splitting strategy to prevent data leakage and minimize training bias, ensuring that spectra from the same animal were exclusively assigned to either the training or test set. Statistical analyses were conducted using one-way analysis of variance (ANOVA) with post-hoc tests applied where appropriate. Significance levels were defined as follows: *p < 0.05 (significant), **p < 0.01 (highly significant), and ***p < 0.001 (extremely significant). Results with p-values > 0.05 were considered statistically non-significant. All measurements of central tendency are reported as mean ± standard deviation (SD).

In analytical techniques for SNR, SNR is calculated as follows:

$$SNR=\frac{s}{\sigma_{s}}$$

$$\sigma_{s}=\sqrt{\frac{\sum[{(y_{i}-y_{a})}^{2}]}{n}}$$

where $s$ represents the signal magnitude, $\sigma_{s}$ is the standard deviation of $s$, $y_{i}$is the signal value of measurement $i$, $y_{a}$is the average signal value, $n$ is the number of repeated measurements, and the summation runs over all repeated measurements $i$.

Model optimization employed a subject-wise partitioning strategy where 20% of subjects were reserved as an independent test set. The remaining 80% of subjects formed the training set, and within this set, hyperparameter optimization was conducted using leave-one-subject-out cross-validation (LOOCV). The XGBoost classifier was implemented within a strict LOOCV framework to ensure generalizability and prevent data leakage. In each LOOCV fold, the spectra of one subject were held out for validation, while the remaining subjects constituted the training set. Feature standardization was performed independently per fold using StandardScaler (fit on the training set, applied to both training and validation sets). Within each training set, a nested 5-fold grid search was conducted to optimize the learning_rate (search range: 0.01–0.3) and gamma (search range: 0–1.0), with classification accuracy as the selection metric. For each fold, the model was instantiated with the fold-specific optimal hyperparameters and the following fixed structural parameters: n_estimators=100, max_depth=5, subsample=0.8, colsample_bytree=0.8, objective='binary: logistic', and random_state=42. Performance metrics (accuracy, sensitivity, specificity, AUC) were computed for each held-out subject and then averaged across all LOOCV folds to report the final model performance. Following LOOCV evaluation, a final model was trained on the entire dataset using the averaged optimal hyperparameters for prospective application. The final model was selected based on its performance on the held-out test sets, ensuring robust external validation. The complete reproducible code is available at: https://github.com/butchresearch/icg-raman-glioblastoma-margins.

Spectral fidelity under varying ICG concentrations was quantitatively assessed by calculating Pearson correlation coefficients between processed spectra from each ICG concentration group (10^-10^ M to 10^-7^ M) and the control group (unlabeled samples). Diagnostic performance was evaluated using standard metrics:

$$Sensitivity=\frac{TP}{TP+FN}$$

$$Specificity=\frac{TN}{TN+FP}$$

$$\mathrm{Accuracy}=\frac{TP+TN}{TP+TN+FP+FN}$$

where $TP$, $TN$, $FP$, and $FN$ represent true positives, true negatives, false positives, and false negatives, respectively. These metrics are reported as point estimates, with model robustness further validated through LOOCV. Inter-method agreement was assessed using Cohen's Kappa statistic, with 95% confidence intervals calculated via non-parametric bootstrapping. Correlation analyses were performed using both Pearson's correlation coefficient (for linear relationships) and Spearman's rank correlation coefficient (for monotonic relationships). The statistical significance of correlation coefficients was determined using two-tailed t-tests. All statistical computations and machine learning implementations were executed in Python 3.9 using established libraries including scikit-learn, SciPy, and StatsModels.


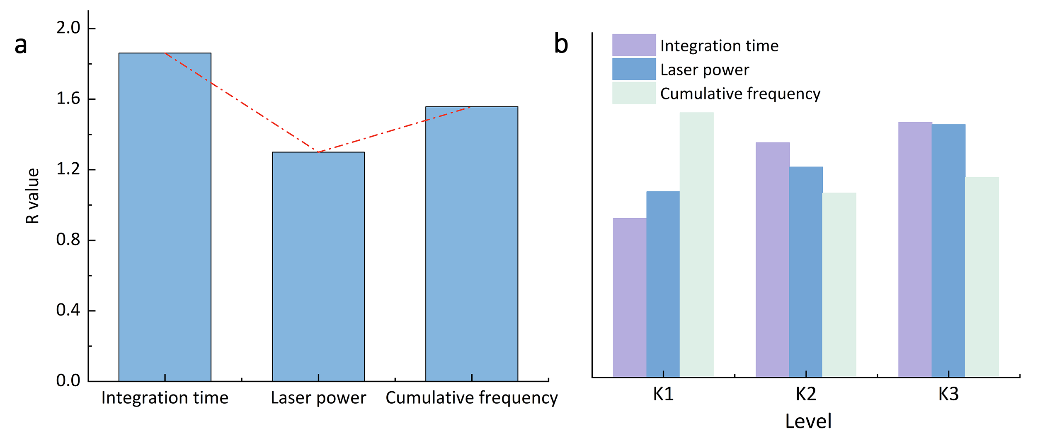


**Figure S1** (a) Range of different factors in orthogonal experiment; (b) Comparison of range values obtained at parameter levels of different factors.


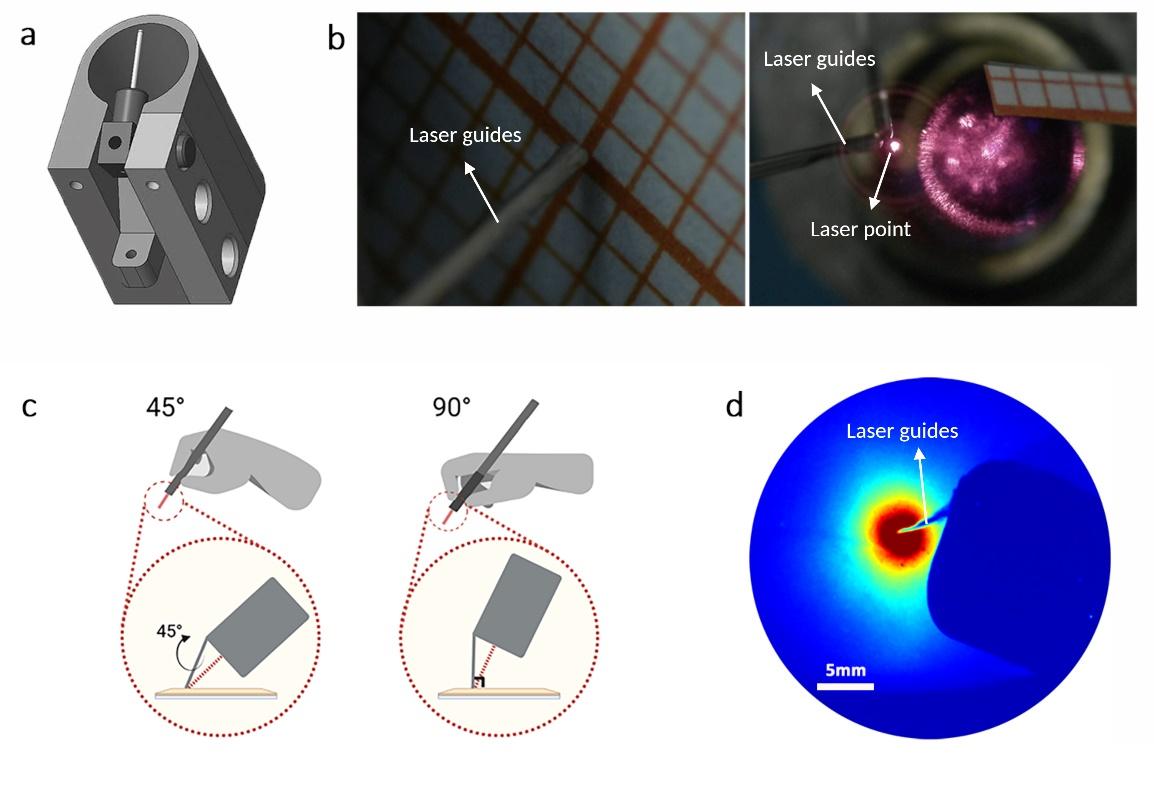


**Figure S2** (a) Illustration of the 3D-printed Raman laser guides model, designed to be securely snapped onto the Raman probe for use; (b) Localized enlargement of the 0.5mm laser guides, red squares with 1mm sides; (c) Schematic diagrams used by the operator at different angles of rotation; (d) Visualization of the effect observed when the laser is activated.


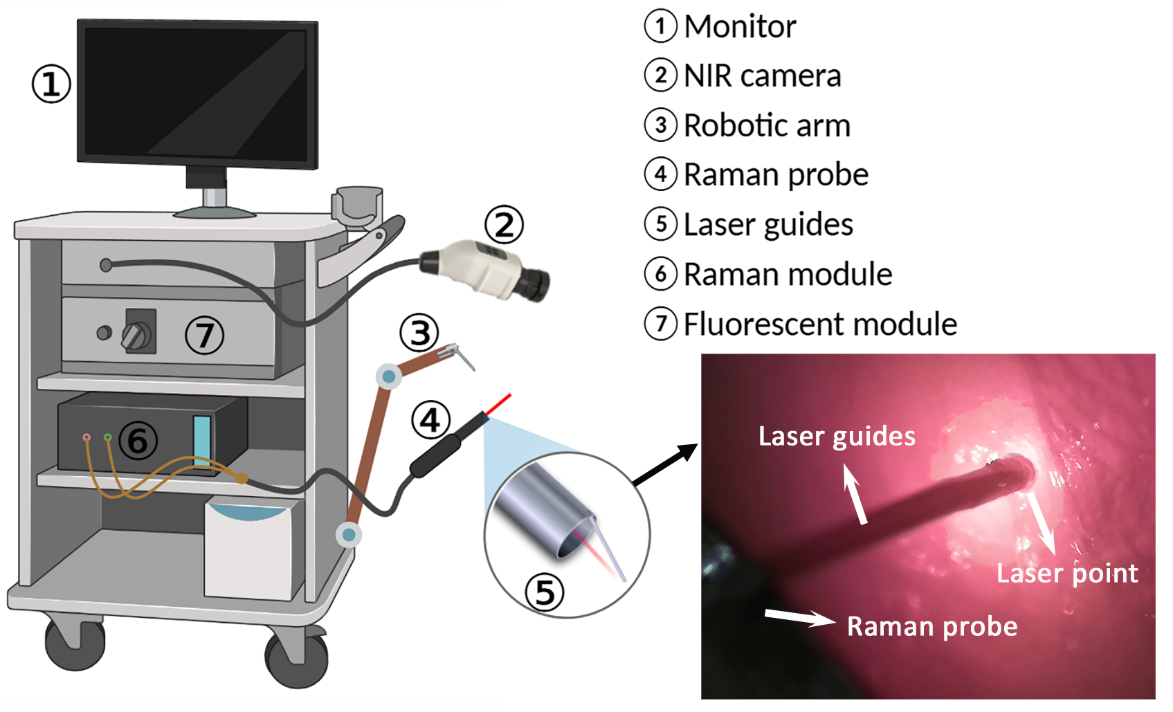


**Figure S3** The surgical imaging system equipment and magnified image of the Raman probe acquisition site.


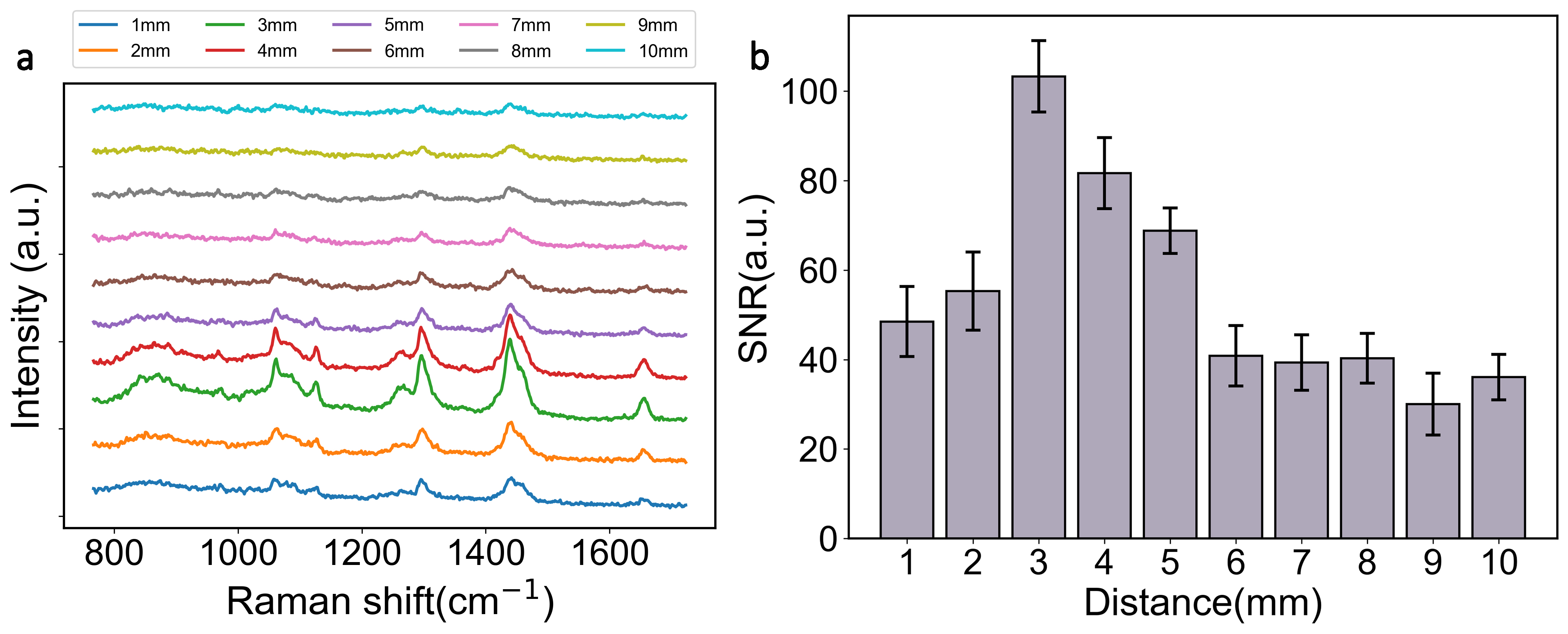


**Figure S4** Spectroscopic characterization of fatty meat at different focusing distances. (a) Raman spectra recorded at focusing distances ranging from 1 to 10 mm; (b) Comparative histogram illustrating the spectral SNR values of spectra obtained at each focusing distance.


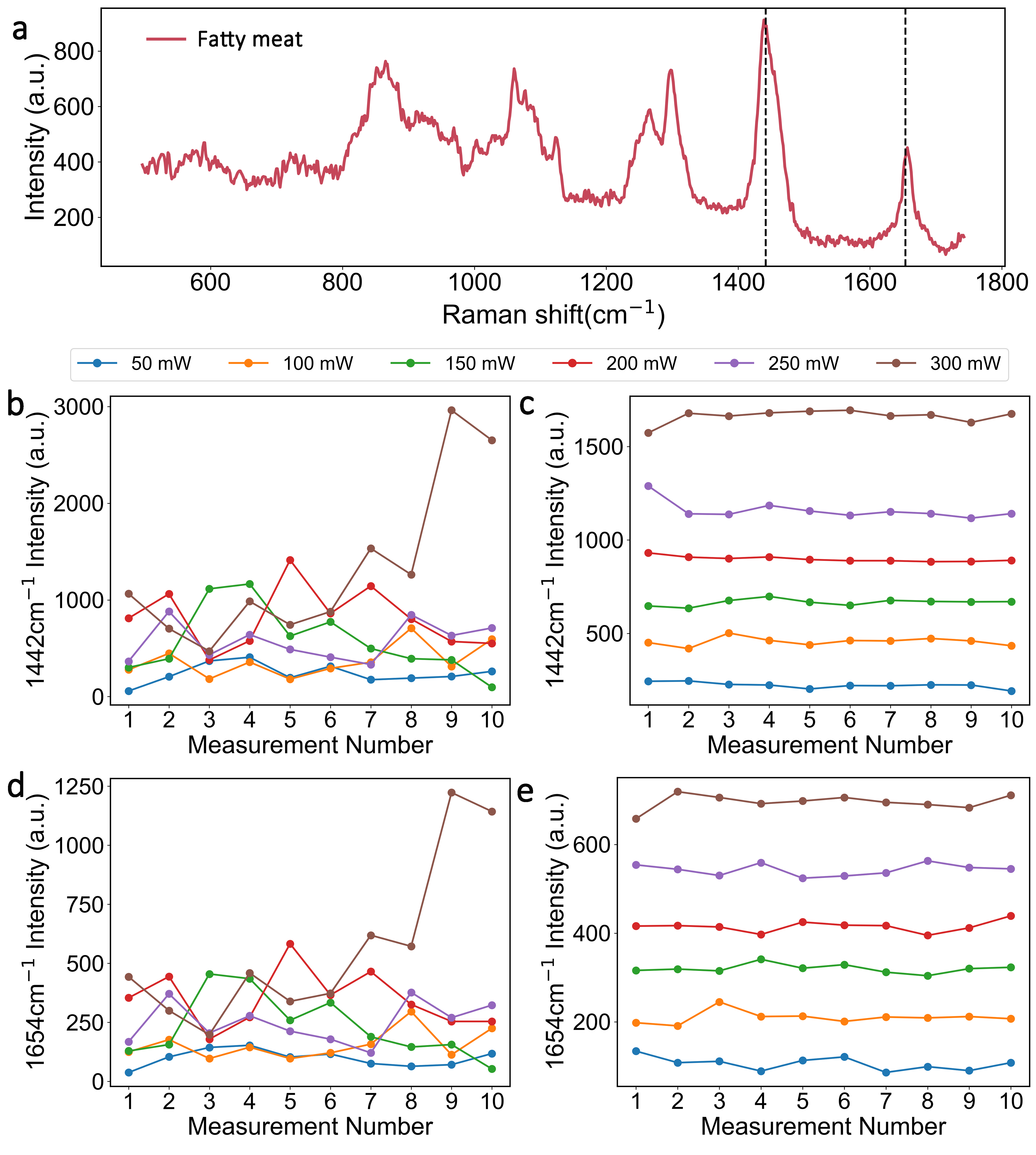


**Figure S5** Raman analysis of fatty tissue using a handheld Raman probe. (a) Representative Raman spectrum of fatty tissue. (b, c) Intensity distributions of the characteristic Raman peak at 1442 cm⁻¹ acquired with 3 s integration at different laser powers: (b) manual handheld operation; (c) measurement with laser guides. (d, e) Corresponding intensity distributions for the peak at 1654 cm⁻¹: (d) manual handheld operation; (e) measurement with laser guides.


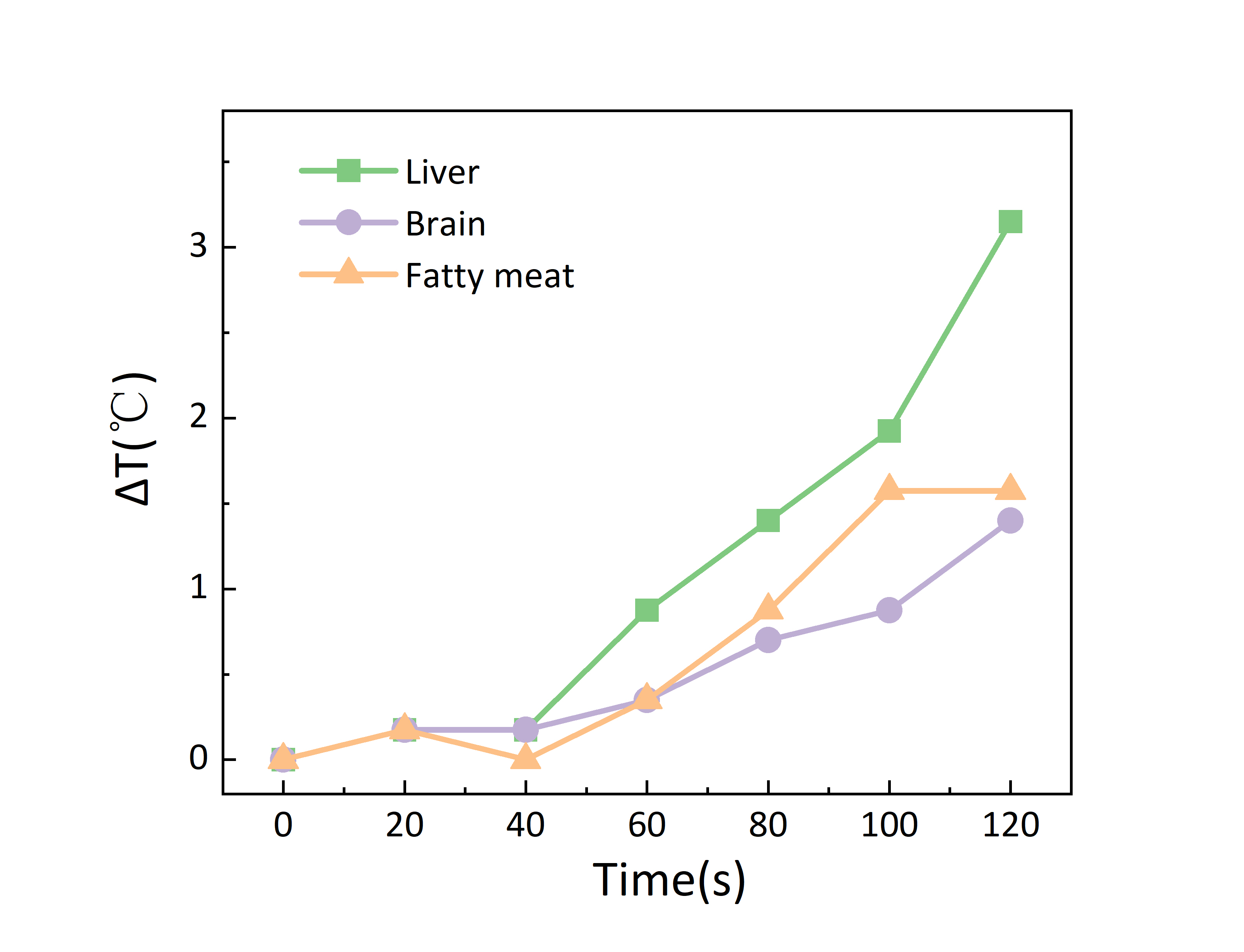


**Figure S6** Curves of temperature-time variation of 250 mw laser excitation on different tissues.


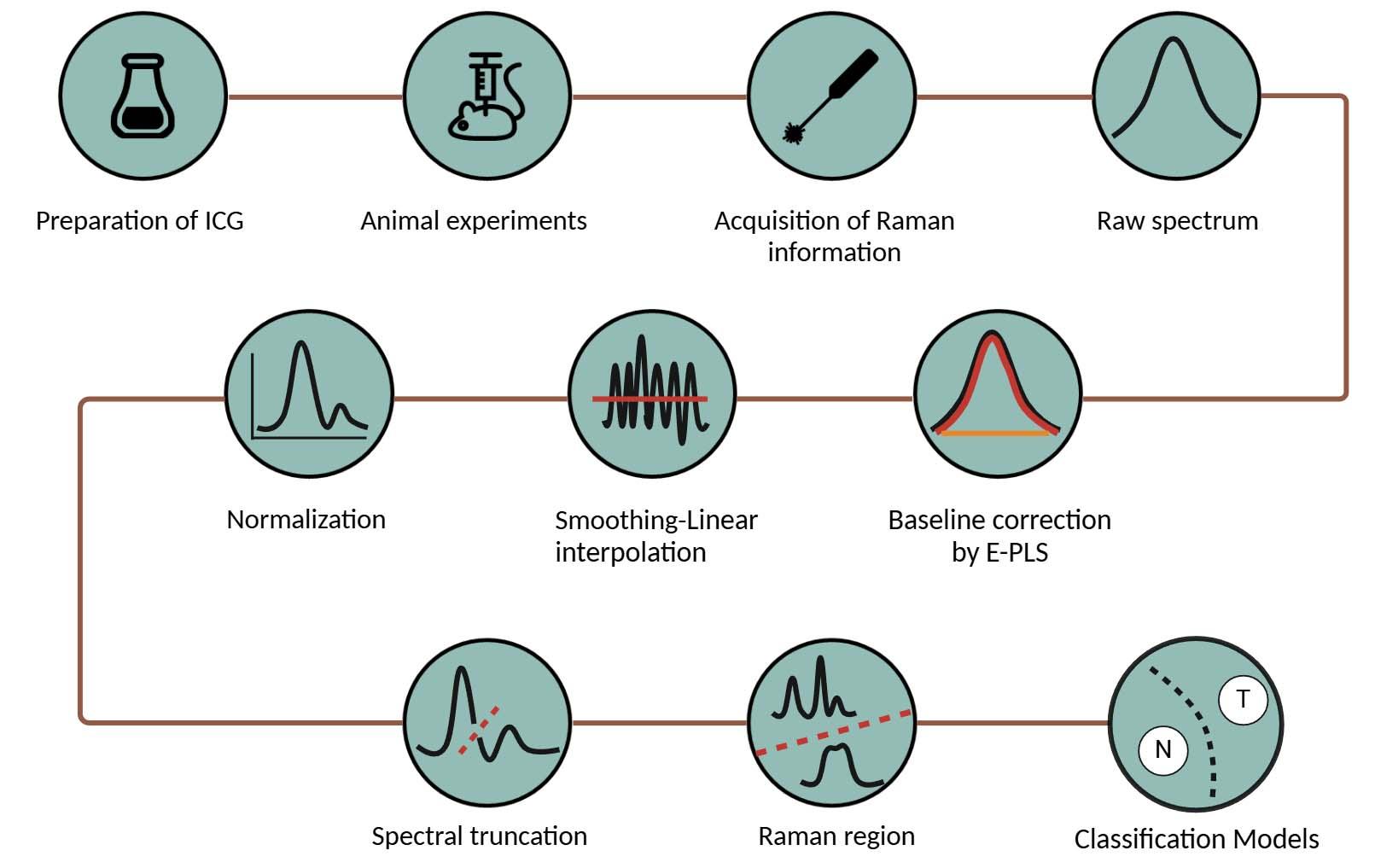


**Figure S7** Detailed overview of the protocol with Raman analysis.


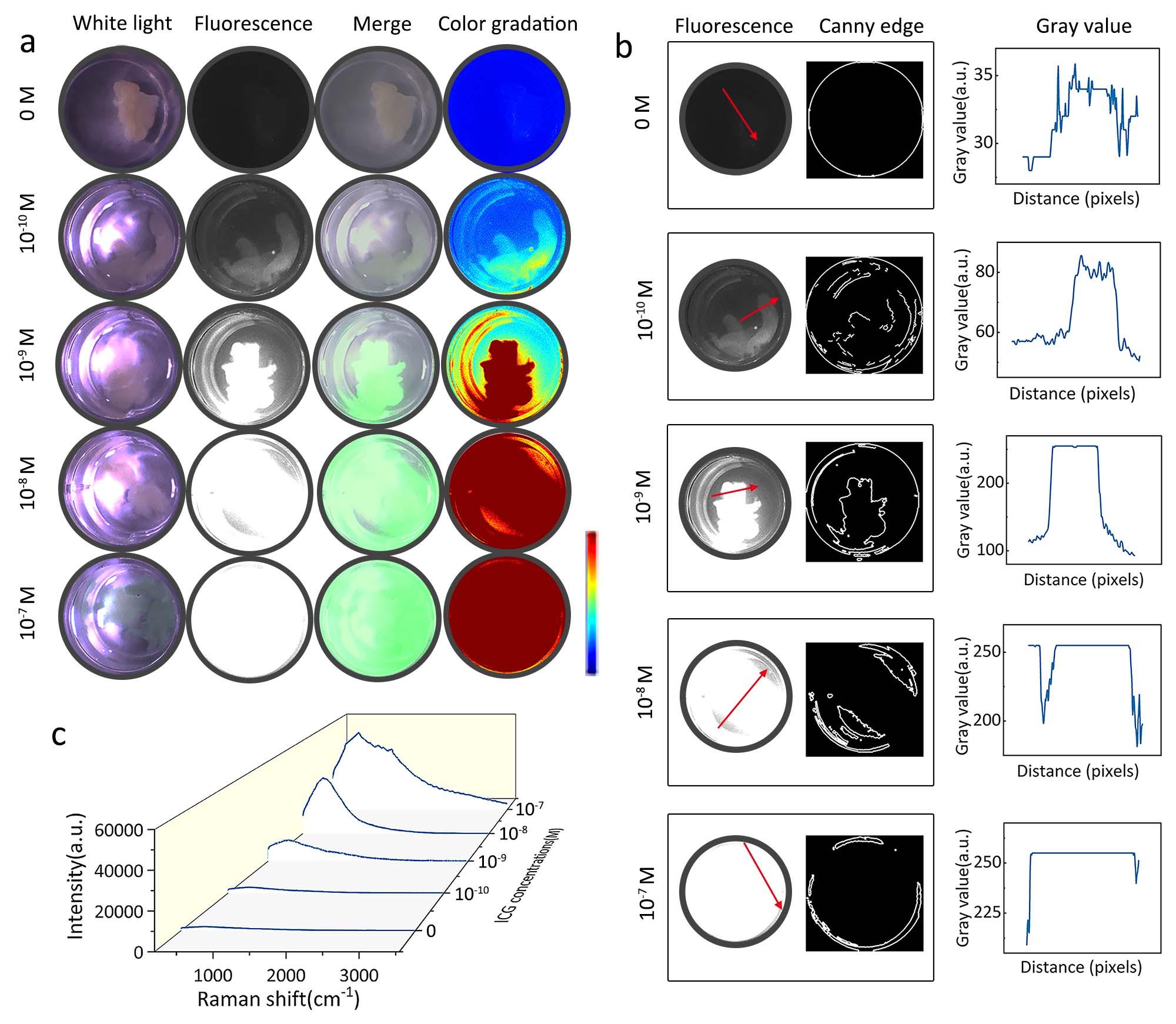


**Figure S8** (a) Imaging of pork with different concentrations of ICG; (b) Canny edge detection of tissue fluorescence images with gray value variation along the line; (c) Original Raman spectra of pork labeled with different concentrations of ICG.


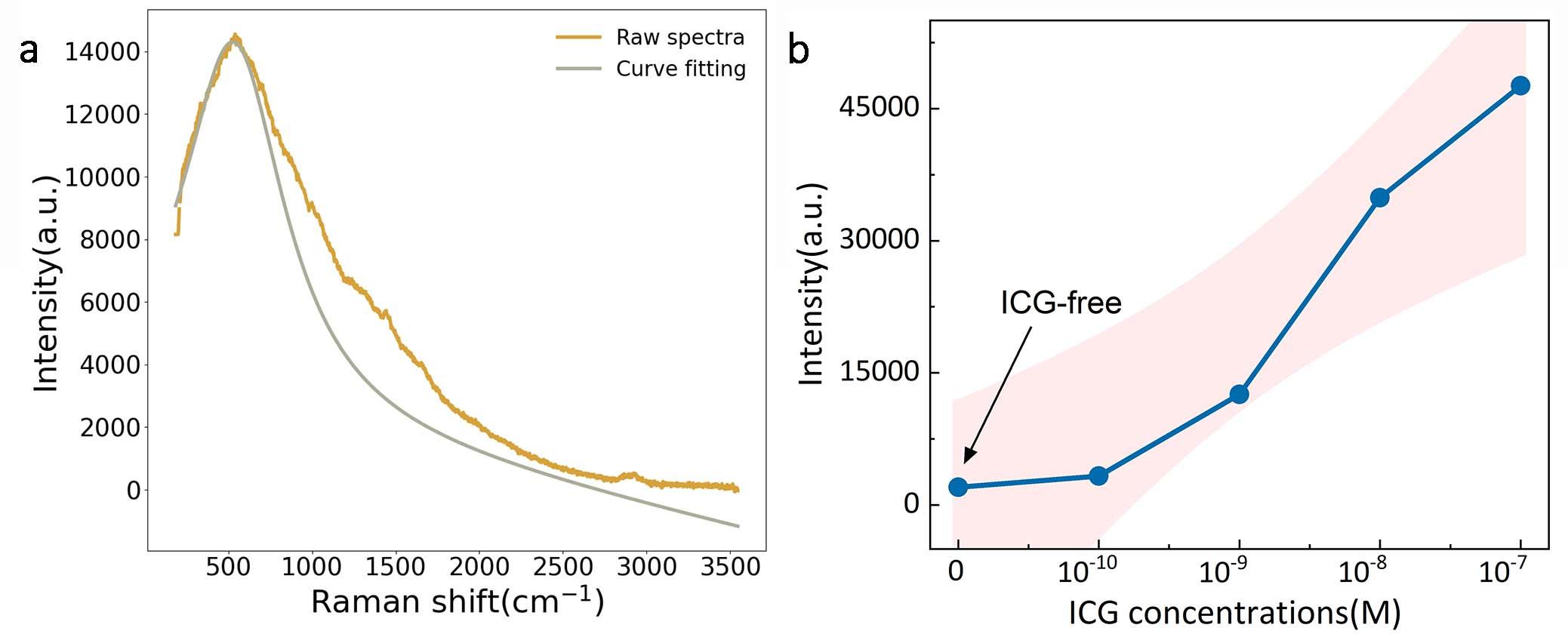


**Figure S9** (a) Fitting of the fluorescence peak at 531 cm^-1^ of the raw spectrum; (b) The value of the 531 cm^-1^ peak fluorescence over time using continuous laser excitation at the same point. The solid line is the mean value and the shading is the standard deviation.


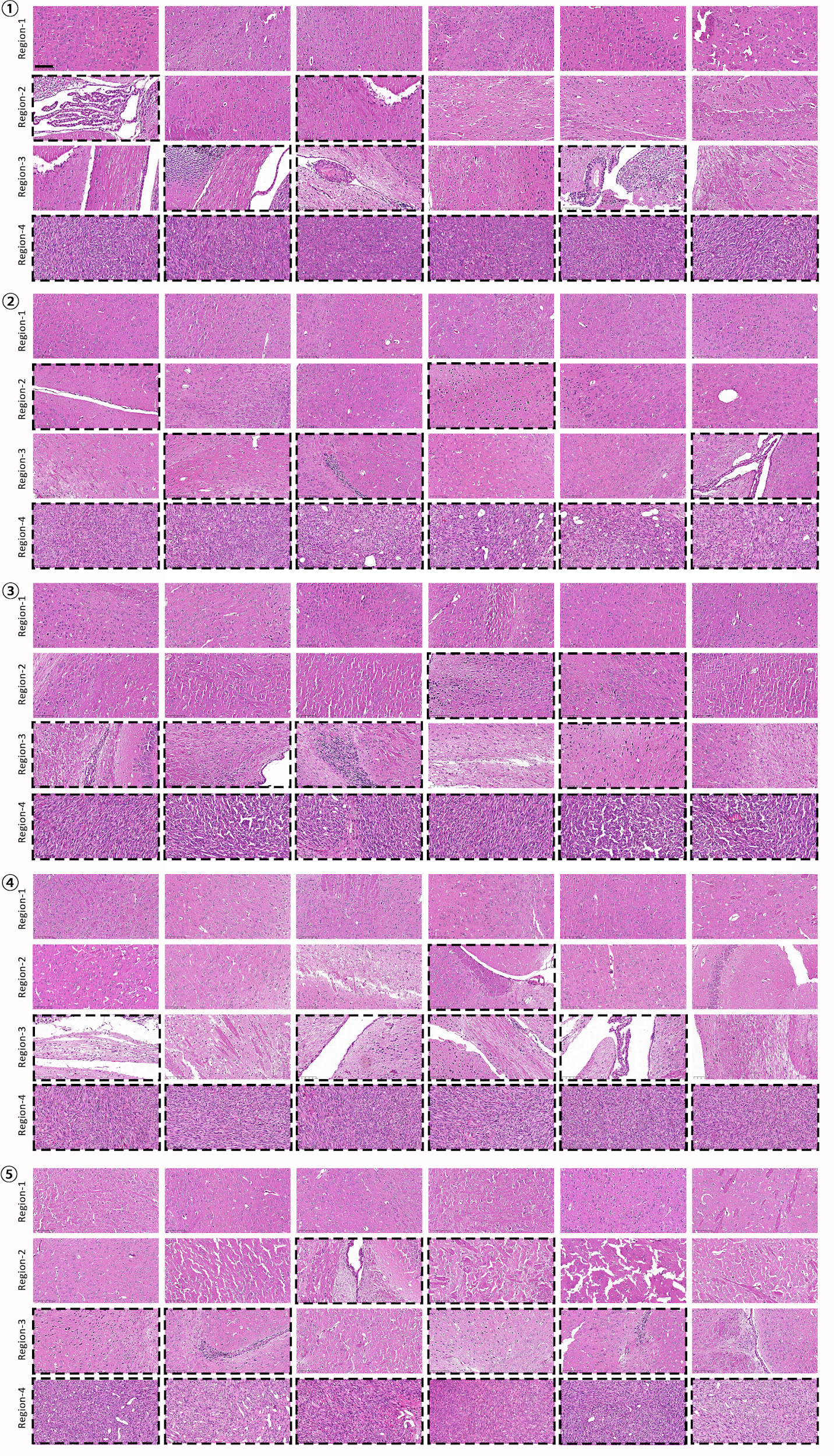


**Figure S10** Pathological results statistics of each group, with tumor tissue in the black box and a scale bar of 100 μm.


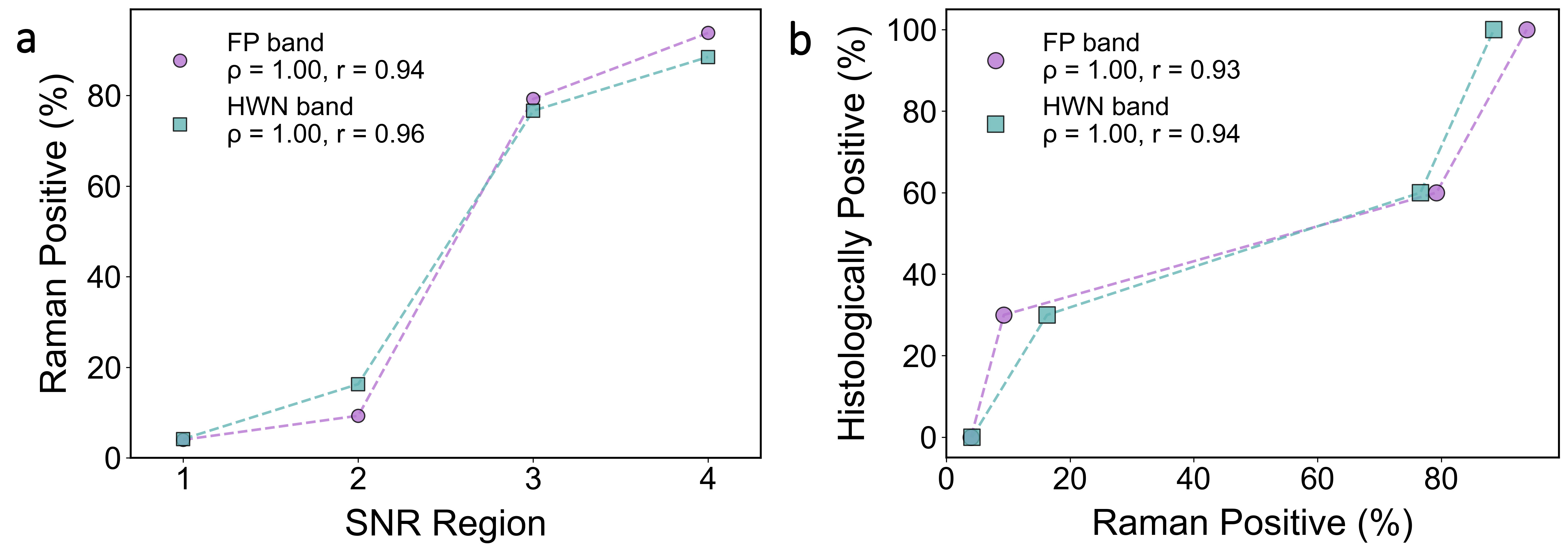


**Figure S11** (a) Classification rate analysis for FP and HWN bands across different SNR regions; (b) Comparison of histologically positive rate and classification rate for FP and HWN bands.


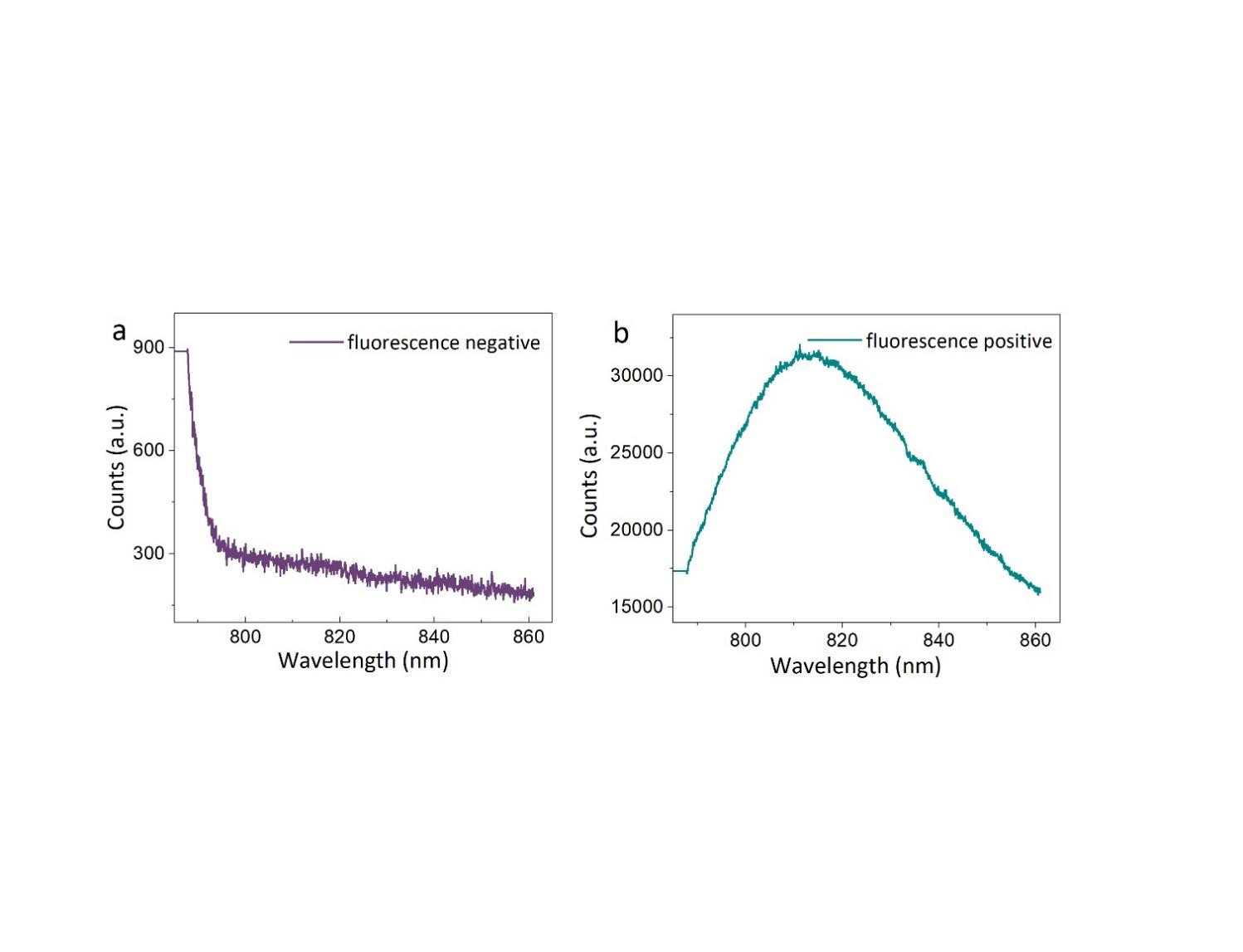


**Figure S12** Fluorescence spectra of fluorescent negatives and positives measured in the fluorescence mode of confocal Raman: (a) fluorescent negative, (b) fluorescent positive.

**
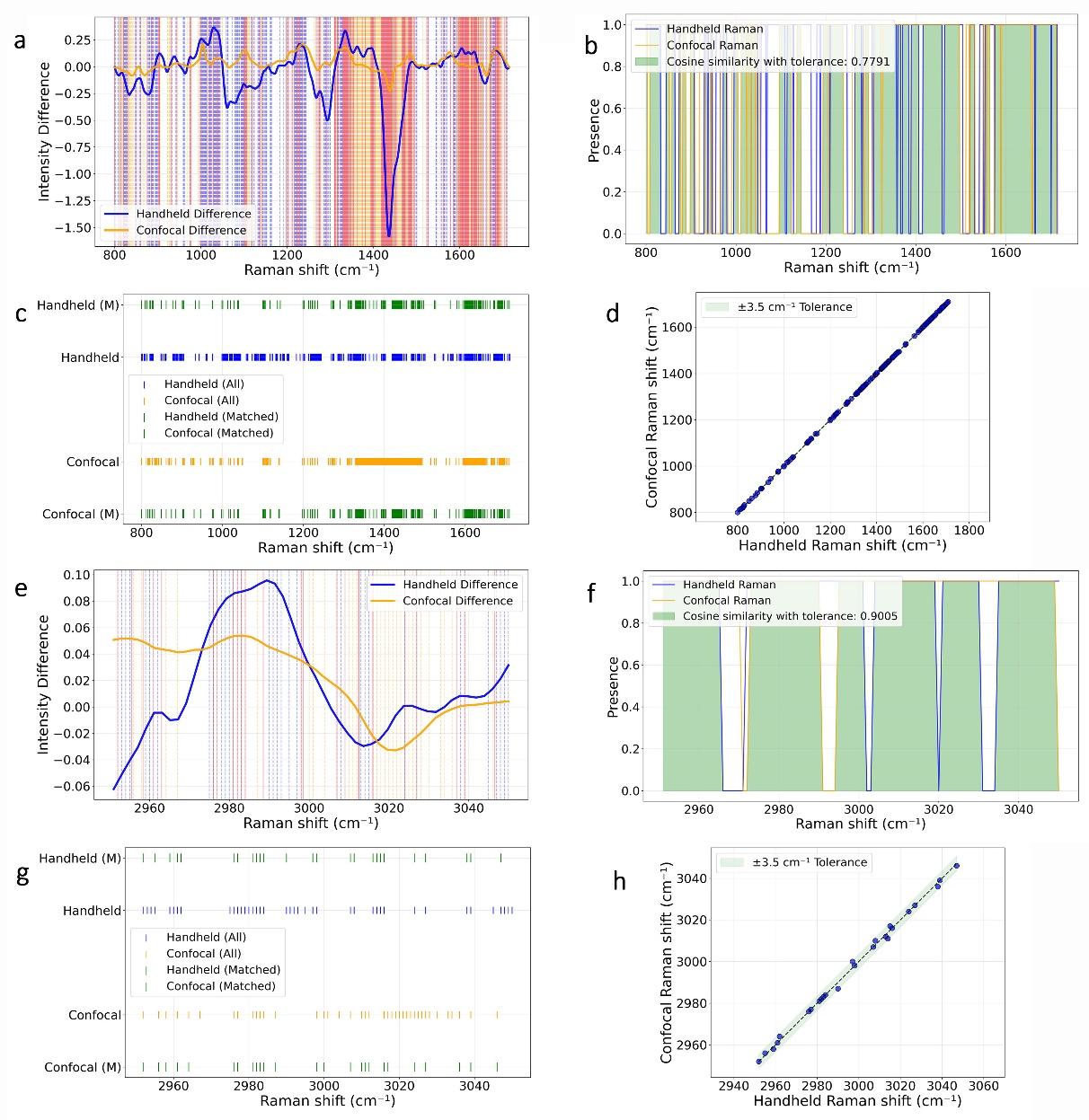
**

**Figure S13 Consistent identification of key spectral features in FP and HWN regions across handheld and confocal Raman platforms.** (a, e) Difference spectra with important features identified by XGBoost for handheld and confocal Raman; (b, f) Feature presence distribution with ±3.5 cm⁻¹ tolerance showing overlap (green) between modalities; (c, g) Distribution of important spectral features across wavelength range with matched features highlighted in green; (d, h) Correlation plot of matched features between platforms showing strong linear relationship within ±3.5 cm⁻¹ tolerance band.

**
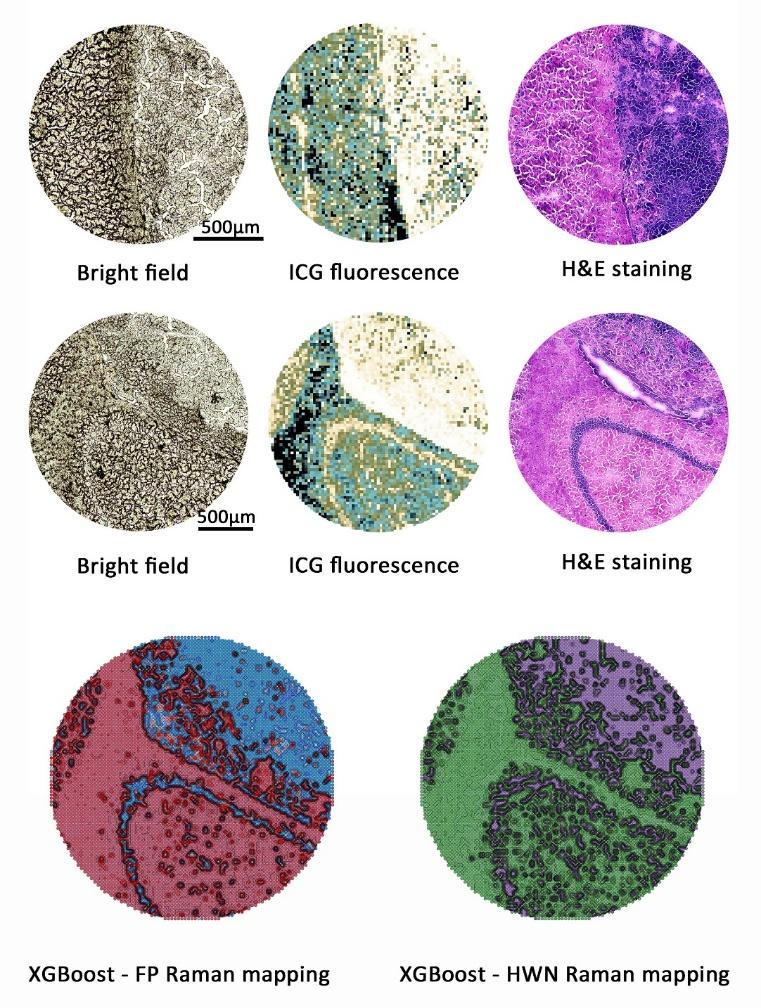
**

**Figure S14** Representative images of the original Raman spectral data, bright-field microscopy, ICG fluorescence, H&E staining, and corresponding tumor probability contour maps in the Raman FP and HWN regions.

**Table S1** Parameters of 3-factor 3-level orthogonal experiment.

| Level | Integration time/s | Laser power/mw | Cumulative frequency |
| --- | --- | --- | --- |
| 1 | 1 | 150 | 1 |
| 2 | 3 | 200 | 3 |
| 3 | 5 | 250 | 5 |

**Table S2** Table of orthogonal tests.

| Serial number | Factors | | | SNR |
| --- | --- | --- | --- | --- |
|  | Integration time/s | Laser power/mw | Cumulative frequency |  |
| 1 | 1 | 150 | 1 | 3.653280506 |
| 2 | 1 | 200 | 3 | 2.814984842 |
| 3 | 1 | 250 | 5 | 2.767874081 |
| 4 | 3 | 150 | 5 | 3.2679842 |
| 5 | 3 | 200 | 3 | 4.021993264 |
| 6 | 3 | 250 | 1 | 6.34373467 |
| 7 | 5 | 150 | 3 | 3.866474704 |
| 8 | 5 | 200 | 1 | 5.378716648 |
| 9 | 5 | 250 | 5 | 5.574105817 |

**Table S3** SNR optimal level screening.

| Serial number | Factors | | |
| --- | --- | --- | --- |
|  | Integration time/s | Laser power/mw | Cumulative frequency |
| K1 | 9.236139429 | 10.78773941 | 15.37573182 |
| K2 | 13.63371213 | 12.21569475 | 10.70345281 |
| K3 | 14.81929717 | 14.68571457 | 11.6099641 |
| Optimum level | K3 | K3 | K1 |
| k1 | 3.078713143 | 3.595913137 | 5.125243941 |
| k2 | 4.544570711 | 4.071898251 | 3.567817603 |
| k3 | 4.939765723 | 4.895238189 | 3.869988033 |
| Extreme deviation R | 1.86105258 | 1.299325052 | 1.557426338 |
| Importance ranking of factors | Integration time＞Cumulative frequency＞Laser power | | |

**Table S4** Common Raman band assignments used in cancer diagnostic studies.

| **Raman shift(cm^−1^)** | **Vibrational mode** | **Assignment** |
| --- | --- | --- |
| 841 | δ(N–CH_2_), ring vibrations | Creatinine |
| 963 | ν(PO_4_^−3^) | Calcium Hydroxyapatite |
| 996 | ν(C–C) ring breathing | Phenylalanine |
| 1049 | ν(C–O), ν(C–N) | Proteins |
| 1158 | C–N and C–C | Proteins, Lipids, Carotenoids |
| 1223 | ν(C–C_6_H_5_) Tyrosine | Phenylalanine |
| 1207 | C–C_6_H_5_ | Phenylalanine, Tryptophan |
| 1261 | ν(C–N), δ(NH) amide III | Proteins, Collagen |
| 1300 | t (C–H) | Fatty Acids |
| 1430 | δ(CH_2_) | Collagen, Lipids |
| 1568 | δ(C=C) | Phenylalanine |
| 1647 | ν(C=C) amide I, α-helix structures | Proteins, Collagen |
| 2842 | ν(CH_2_) | Lipids |
| 2856 |  | Lipids |
| 2875 | ν(CH_2_) | Proteins, Lipids |
| 2940 | ν(CH_3_) | Proteins, Lipids |

**Table S5** The confusion matrix of fluorescent regions with pathology for different thresholds.

| Region-1 | | |
| --- | --- | --- |
|  | Fluorescence positive | Fluorescence negative |
| Pathologically positive | 57 | 0 |
| Pathologically negative | 63 | 0 |
| Region-2 | | |
|  | Fluorescence positive | Fluorescence negative |
| Pathologically positive | 57 | 0 |
| Pathologically negative | 33 | 30 |
| Region-3 | | |
|  | Fluorescence positive | Fluorescence negative |
| Pathologically positive | 48 | 9 |
| Pathologically negative | 12 | 51 |
| Region-4 | | |
|  | Fluorescence positive | Fluorescence negative |
| Pathologically positive | 30 | 27 |
| Pathologically negative | 0 | 63 |

**Table S6** Diagnostic indices of fluorescence region compared to pathology at different thresholds in Ex Vivo.

| **Threshold** | Region-1 | Region-2 | Region-3 | Region-4 |
| --- | --- | --- | --- | --- |
| **Sensitivity** | 100% | 100% | 84.2% | 52.6% |
| **Specificity** | 0 | 47.6% | 81.0% | 100% |
| **Accuracy** | 47.5% | 72.5% | 82.5% | 76.7% |

**Table S7** The confusion matrix of fluorescent regions with Raman band prediction classification results for different thresholds in Ex Vivo experiments.

|  | FP band | | HWN band | |
| --- | --- | --- | --- | --- |
| Threshold 1 | Fluorescence positive | Fluorescence negative | Fluorescence positive | Fluorescence negative |
| Raman positive | 163 | 0 | 163 | 0 |
| Raman negative | 118 | 0 | 118 | 0 |
|  | FP band | | HWN band | |
| Threshold 2 | Fluorescence positive | Fluorescence negative | Fluorescence positive | Fluorescence negative |
| Raman positive | 162 | 1 | 157 | 3 |
| Raman negative | 63 | 55 | 62 | 53 |
|  | FP band | | HWN band | |
| Threshold 3 | Fluorescence positive | Fluorescence negative | Fluorescence positive | Fluorescence negative |
| Raman positive | 97 | 4 | 165 | 11 |
| Raman negative | 21 | 159 | 19 | 82 |
|  | FP band | | HWN band | |
| Threshold 4 | Fluorescence positive | Fluorescence negative | Fluorescence positive | Fluorescence negative |
| Raman positive | 96 | 67 | 94 | 69 |
| Raman negative | 4 | 114 | 6 | 112 |

**Table S8** Diagnostic indices of gliomas by fluorescent regions and Raman bands at different thresholds in Ex Vivo.

|  | Region-1 | Region-2 | Region-3 | Region-4 |
| --- | --- | --- | --- | --- |
| FP band | | | | |
| Sensitivity | 100% | 99.4% | 96.0% | 58.9% |
| Specificity | 0 | 46.6% | 88.3% | 96.6% |
| Accuracy | 58.0% | 77.2% | 91.2% | 76.1% |
| HWN band | | | | |
| Sensitivity | 100% | 98.1% | 93.8% | 57.7% |
| Specificity | 0 | 46.1% | 81.2% | 94.9% |
| Accuracy | 58.0% | 76.7% | 85.4% | 74.7% |

**Table S9** The confusion matrix of fluorescent regions with Raman band prediction classification results for different thresholds in In Vivo experiments.

|  | FP band | | HWN band | |
| --- | --- | --- | --- | --- |
| Threshold 1 | Fluorescence positive | Fluorescence negative | Fluorescence positive | Fluorescence negative |
| Raman positive | 173 | 0 | 170 | 0 |
| Raman negative | 110 | 0 | 113 | 0 |
|  | FP band | | HWN band | |
| Threshold 2 | Fluorescence positive | Fluorescence negative | Fluorescence positive | Fluorescence negative |
| Raman positive | 171 | 2 | 166 | 4 |
| Raman negative | 62 | 48 | 67 | 46 |
|  | FP band | | HWN band | |
| Threshold 3 | Fluorescence positive | Fluorescence negative | Fluorescence positive | Fluorescence negative |
| Raman positive | 167 | 6 | 159 | 11 |
| Raman negative | 23 | 87 | 31 | 82 |
|  | FP band | | HWN band | |
| Threshold 4 | Fluorescence positive | Fluorescence negative | Fluorescence positive | Fluorescence negative |
| Raman positive | 103 | 67 | 100 | 70 |
| Raman negative | 7 | 106 | 13 | 100 |

**Table S10** Diagnostic indices of gliomas by fluorescent regions and Raman bands at different thresholds in In Vivo.

|  | Region-1 | Region-2 | Region-3 | Region-4 |
| --- | --- | --- | --- | --- |
| FP band | | | | |
| Sensitivity | 100% | 98.8% | 96.5% | 60.6% |
| Specificity | 0 | 43.6% | 79.1% | 93.8% |
| Accuracy | 61.1% | 77.4% | 89.7% | 73.8% |
| HWN band | | | | |
| Sensitivity | 100% | 97.6% | 93.5% | 58.8% |
| Specificity | 0 | 40.7% | 72.6% | 88.5% |
| Accuracy | 60.0% | 74.9% | 85.2% | 70.7% |

**Table S11.** Performance comparison between a Raman-only paradigm and this work.

| **Comparison items** | **Raman-only** | **This work** |
| --- | --- | --- |
| Acquisition strategy | Exhaustive, untargeted point sampling | Fluorescence-guided targeted sampling |
| Time per raman spectrum | ~3 s | ~3 s |
| Time for field assessment | Impractical for large areas (e.g., ~600s for 200 points) | ~60s (real-time fluorescence + targeted raman on 20 points) |
| Tissue classification accuracy (per-spectrum) | 85–90% (xgboost model performance) | 85–90% (same model applied to targeted points) |
| False positive rate guidance | N/A | Actively reduced. Corrects icg false positives |
| Spatial accuracy of tumor boundary | Not applicable intraoperatively; a single comprehensive scan requires hours. | Validated as high. Raman boundary matches histology |
